# Analysis of biomarkers in speculative CNS-enriched extracellular vesicles for parkinsonian disorders: a comprehensive systematic review and diagnostic meta-analysis

**DOI:** 10.1007/s00415-023-12093-3

**Published:** 2023-12-16

**Authors:** Hash Brown Taha, Aleksander Bogoniewski

**Affiliations:** 1 Department of Integrative Biology and Physiology, University of California Los Angeles, Los Angeles, CA USA; 2 Department of Molecular and Medical Pharmacology, University of California Los Angeles, Los Angeles, CA USA

**Keywords:** L1CAM, Exosome, Parkinson’s disease, Movement disorders, Diagnosis, Alpha-synuclein

## Abstract

**Background and objective:**

Parkinsonian disorders, including Parkinson's disease (PD), multiple system atrophy (MSA), dementia with Lewy bodies (DLB), progressive supranuclear palsy (PSP), and corticobasal syndrome (CBS), exhibit overlapping early-stage symptoms, complicating definitive diagnosis despite heterogeneous cellular and regional pathophysiology. Additionally, the progression and the eventual conversion of prodromal conditions such as REM behavior disorder (RBD) to PD, MSA, or DLB remain challenging to predict. Extracellular vesicles (EVs) are small, membrane-enclosed structures released by cells, playing a vital role in communicating cell-state-specific messages. Due to their ability to cross the blood–brain barrier into the peripheral circulation, measuring biomarkers in blood-isolated speculative CNS enriched EVs has become a popular diagnostic approach. However, replication and independent validation remain challenging in this field. Here, we aimed to evaluate the diagnostic accuracy of speculative CNS-enriched EVs for parkinsonian disorders.

**Methods:**

We conducted a PRISMA-guided systematic review and meta-analysis, covering 18 studies with a total of 1695 patients with PD, 253 with MSA, 21 with DLB, 172 with PSP, 152 with CBS, 189 with RBD, and 1288 HCs, employing either hierarchical bivariate models or univariate models based on study size.

**Results:**

Diagnostic accuracy was moderate for differentiating patients with PD from HCs, but revealed high heterogeneity and significant publication bias, suggesting an inflation of the perceived diagnostic effectiveness. The bias observed indicates that studies with non-significant or lower effect sizes were less likely to be published. Although results for differentiating patients with PD from those with MSA or PSP and CBS appeared promising, their validity is limited due to the small number of involved studies coming from the same research group. Despite initial reports, our analyses suggest that using speculative CNS-enriched EV biomarkers may not reliably differentiate patients with MSA from HCs or patients with RBD from HCs, due to their lesser accuracy and substantial variability among the studies, further complicated by substantial publication bias.

**Conclusion:**

Our findings underscore the moderate, yet unreliable diagnostic accuracy of biomarkers in speculative CNS-enriched EVs in differentiating parkinsonian disorders, highlighting the presence of substantial heterogeneity and significant publication bias. These observations reinforce the need for larger, more standardized, and unbiased studies to validate the utility of these biomarkers but also call for the development of better biomarkers for parkinsonian disorders.

**Supplementary Information:**

The online version contains supplementary material available at 10.1007/s00415-023-12093-3.

## Introduction

Parkinsonian disorders comprise a group of neurodegenerative conditions sharing motor symptoms, such as slow movement (bradykinesia), stiffness (rigidity), and shaking (tremor). Parkinson's disease (PD) is the most common among these conditions [28]. Other less frequent but clinically important parkinsonian disorders include multiple system atrophy (MSA), dementia with Lewy bodies (DLB), progressive supranuclear palsy (PSP), and corticobasal syndrome (CBS) [2]. While these disorders differ in the type of protein, cell type, and brain region afflicted, they are often misdiagnosed by neurologists due to symptom overlap, especially in early stages [3, 31, 34]. Moreover, currently, there is no concrete method to precisely ascertain the timing, progression, and specific outcomes of prodromal conditions like REM behavior disorder (RBD) and pure autonomic failure (PAF) [8, 9].

Misdiagnosis not only negatively impacts patient prognosis, potentially leading to inappropriate treatments and worsening health outcomes, but also exacerbates emotional distress, intensifying feelings of uncertainty and anxiety about their health conditions, and impacts appropriate patient stratification in clinical trials. This lack of reliable diagnostic tools also obstructs efforts to assess disease-modifying treatments during the prodromal stages, a critical period where majority of neuronal death occurs [6].

Extracellular vesicles (EVs) are tiny, bi-lipid membrane-enclosed structures released by cells, which play vital roles in facilitating communication among cells and regulating various bodily processes. Unlike living cells, EVs do not replicate and serve as carriers of biological cargo, enabling the exchange of molecular information and contributing to intercellular signaling. They contain a diverse array of biomolecules, including proteins, lipids, and nucleic acids, which mirror the condition of the originating cell [10]. Due to their ability to traverse the blood–brain barrier to the peripheral circulation [38], speculative central nervous nervous system (CNS)-enriched EVs may provide a unique insight into the brain's biochemical processes, enabling the investigation of CNS functions and the identification of potential biomarkers in neurodegenerative conditions such as parkinsonian disorders [13].

As potential carriers of cell-state-specific information from the CNS to the peripheral circulation, speculative CNS-enriched EVs have emerged as a possible tool for minimally invasive diagnostic and therapeutic strategies in parkinsonian disorders. Many groups have quantified biomarkers in speculative CNS-enriched EVs for the differential diagnosis of these disorders from one another and/or from healthy controls (HCs) [13]. Despite this, there has been consistent failure in independent validations, replication, and differing outcomes even when the same methodology is employed.

A recent meta-analysis suggested that the combined concentration of α-synuclein (α-syn) in speculative neuronal and oligodendroglial EVs (nEVs and oEVs, respectively) may be higher in patients with PD in comparison to HCs, CBS, and PSP [41]. These elevated concentrations could potentially be utilized to develop a diagnostic test for these diseases. However, the meta-analysis did not compare the diagnostic accuracy of tests utilizing biomarkers in speculative CNS-enriched EVs, which include α-syn combined with other biomarkers.

Our goal is to expand upon previous findings by conducting a meta-analysis of diagnostic accuracy using studies attempting to differentiate either prodromal or established parkinsonian disorders from each other or from HCs, using biomarkers in speculative CNS-enriched EVs. We use the term “speculative CNS-enriched EVs” for two key reasons. Firstly, current research has yet to conclusively demonstrate that these enriched EVs originate specifically from the brain. This uncertainty is compounded by the fact that the markers used to perform CNS enrichment are also found on other cell types, or even in soluble forms [26], which have been shown to cross-react with the antibodies used for biomarker quantification. Secondly, the integrity of these EVs as purely CNS-originating is questionable. EVs are known to be absorbed and recycled by various cells through different mechanisms, even if they initially come from the CNS [10]. This process of uptake and rerelease further obscures their original CNS origin.

## Methodology

We performed a systematic review and meta-analysis according to the guidelines outlined in the Preferred Reporting Items for Systematic Reviews and Meta-Analyses Protocols (PRISMA). Our research exclusively utilized anonymized data, with no collection of personal information or involvement of human subjects, thus obviating the need for ethical approval. The study protocol was not registered.

### Standard protocol approvals, registrations, and patient consents

Standard protocol approvals, registration, and patient consents are not applicable to this meta-analysis.

### Data sources and search strategy

We performed a thorough search for relevant articles using specific search terms related to PD and parkinsonian disorders. The search was conducted in two databases (PUBMED and EMBASE) and covered articles published from the inception of the databases until Sept 29, 2023. The search terms we used included combinations of “Parkinson's disease OR multiple system atrophy OR Lewy body dementia OR corticobasal syndrome OR progressive supranuclear palsy” AND “Extracellular Vesicle OR exosome” AND “Diagnosis”. We manually examined the reference lists of eligible studies and conducted thorough literature reviews to identify suitable studies for inclusion. Any discrepancies in the selection of articles were resolved through discussions. The comprehensive search strategy can be accessed in Table S1.

### Eligibility criteria

The eligible studies included in our analysis focused on assessing biomarkers in speculative CNS-enriched EVs isolated from cerebrospinal fluid, plasma, serum, urine, or saliva in patients with PD along with at least one of the following diseases: MSA, DLB, PSP, CBS, RBD, PAF, or HCs. The studies must have included a receiver operating characteristic (ROC) analysis and provided sensitivity, specificity, area under curve (AUC), and sample size. We excluded studies that used animals or cell lines, studies that did not include the specified diseases, and studies that did not report the sample size. We excluded studies that have used general EVs as they have been reviewed elsewhere [42]. If sensitivity, specificity, or sample size were not included in the study, we contacted the authors to obtain the missing information. For studies that included longitudinal measurements or treatment interventions, we only considered the baseline assessments. For studies that included discovery/training and validation ROC models, we only considered the validation model. In cases where more than 2 ROC models existed, we chose the model with the best AUC for reporting. In three studies [1, 16, 24], two models performed similarly, and we included both models. In one study [35], there were only two models, but we excluded the one with AUC close to 0.50, indicating no accuracy.

### Risk of bias assessment

The quality and the risk of bias of all eligible studies were evaluated using the Quality Assessment for Diagnostic Accuracy Studies (QUADAS-2) criteria [47]. The assessment was carried out by independent researchers (HBT and AB), and any disagreements were resolved through discussion until a consensus was reached. Additional details regarding the quality assessment can be found in Table S2.

### Data synthesis and statistics

In this study, we chose a hierarchical summary ROC (HSROC) and a bivariate model [20, 30] utilizing a random effect with a restricted maximum likelihood estimation method in analyses where the number of studies was > 3. This approach allows a comprehensive assessment of the diagnostic accuracy measures, accounting for both within-study and between-studies variability as well as the inherent negative correlation between sensitivities and specificities across studies. In cases where the number of studies was ≤ 3, we utilized a univariate model as the parameters in the bivariate model are not recommended when there are only a few studies [45]. Detailed information regarding the HSROC and bivariate models is described elsewhere [42]

In addition, we utilized informative graphical representations, including crosshair plots, which integrate both ROC curves and forest plot means. These visualizations allow us to simultaneously examine the bivariate relationship between sensitivity and false-positive rate (FPR or 1-specificity) while assessing the degree of heterogeneity across studies. Notably, wider crosshairs on the plot indicate a larger sample size, reflecting the level of precision and reliability in the estimates. The ROC ellipse plot visually represents the estimated uncertainty of the pair (sensitivity, FPR) in logit ROC space using confidence regions. The ellipses in the plot symbolize the variability of the sensitivity and FPR estimates, providing an indication of their statistical uncertainty.

The summary ROC curve utilized both the dotted means obtained from the bivariate model with its corresponding confidence interval as well as the summary line obtained from the HSROC model [32], which describes the relationship between the mean sensitivity and specificity. In this meta-analysis, when significant heterogeneity is present, the summary line provides more informative results compared to the point means of sensitivities and specificities, as it comprehensively takes into account the heterogeneity across the included studies [45]. The accuracy of the test increases as the point summary of sensitivities/specificities and the summary line approaches the upper left corner.

Funnel plots, Begg’s rank correlation, Egger’s and Deek’s regression tests, and trim-and-fill method [36] were used to evaluate publication bias [21].

## Results

**Fig. 1:**
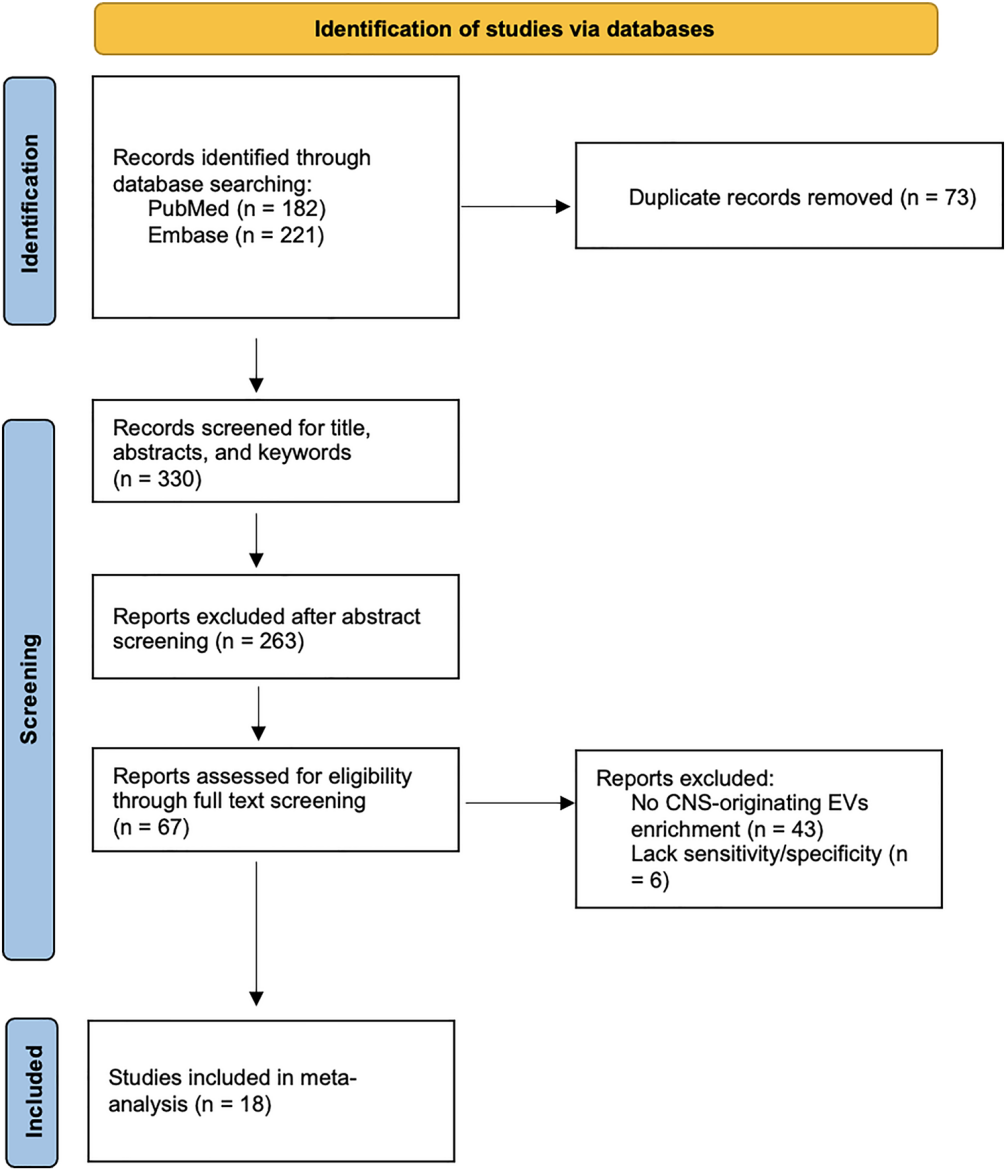
PRISMA flow diagram for inclusion of selected studies

The systematic search identified 403 studies of which 73 duplicated studies were removed. After title and abstract screening of 330 studies, 67 studies were considered potentially eligible (Fig. 1). After full-text screening, 49 studies were excluded. Forty-three of those studies did not enrich for speculative CNS-enriched EVs. Five studies enriched for speculative CNS-enriched EVs [4, 19, 22, 23, 27] but did not include information for sensitivity and specificity from the diagnostic test. One study was excluded because it included preliminary data [14]. All authors were contacted to obtain the missing information.

In total, the meta-analysis included 18 studies [1, 5, 11, 16–18, 24, 25, 35, 37–39, 43, 46, 48–50, 52] with 1695 patients with PD, 253 with MSA, 21 with DLB, 172 with PSP, 152 with CBS, 189 with RBD, and 1288 HCs (Table 1). Using biomarkers in speculative CNS-enriched EVs, most studies attempted to differentiate patients with PD from HCs (*n* = 16, 88.8%). Six studies attempted to differentiate patients with PD from MSA [11, 16, 17, 43, 46, 49] while two studies aimed to differentiate patients with PD from PSP and CBS [16, 24]. One study attempted to differentiate patients with PD from frontotemporal dementia (FTD), PSP and CBS [17]. Three studies aimed to differentiate patients with MSA from HCs [11, 43, 49] or patients with RBD from HCs [17, 35, 48]. Most studies utilized biomarkers in speculative nEVs (*n* = 16, 88.8%). Only three studies used speculative oEVs [49,11,43] while one study used speculative astrocyte EVs (aEVs) [46] . To quantify biomarkers in speculative CNS-enriched EVs, the included studies utilized bead-based arrays such as Luminex [38, 49] and single-molecule array (Simoa) [37, 52], enzyme-linked immunosorbent assay (ELISA) [50, 5, 39, 1,24], electrochemiluminescence ELISA (ECLIA) [25, 18, 17, 16,11, 43], western blots (WB) [48], flow-cytometry [46], or an in-house electrochemical assay [35].

**Table 1:** Demographic characteristics of patients with parkinsonian disorders or healthy controls (HC) included in the meta-analysis

Study (first author, year)	Preanalytical factors Anticoagulant molecule: Platelet-depletion (Y/N) Coagulation factor depletion (Y/N)	EV isolation method	CNS-EV antibody	EV confirmation method	EV lysis method	EV frozen (Y/N)	# PD	# MSA	# DLB	# PSP	# CBS	# RBD	# HC	Age (years)	Female (%)	Disease Duration (years)	HY scale	UPDRS III	MMSE	MoCA
*Plasma*																				
Shi et al. 2014 [38]	EDTA Y (centrifuged at 3200* g* for 15 min at 4 °C) N	2000* g* for 15 min to remove cell debris followed by 12,000* g* for 30 min to remove larger EVs/particles followed by direct IP	L1CAM (clone UJ127)	TEM WB for Alix and L1CAM	1% Triton X-100	N	267	0	0	0	0	0	215	PD: 66.3 ± 9.1 HC: 65.7 ± 9.1	PD: 44.6% HC: 46.0%	PD: 9.6 ± 6.6	PD: 2.4 ± 0.7	PD: 28.4 ± 12.6	PD: 28.0 ± 2.6	NR
Shi et al. 2016 [37]	EDTA Y (centrifuged at 3200* g* for 15 min at 4 °C) N	2000* g* for 15 min to remove cell debris followed by 12,000* g* to remove larger EVs/particless for 30 min followed by direct IP	L1CAM (clone UJ127)	TEM WB for Alix and L1CAM	1% Triton X-100	N	91	0	0	0	0	0	106	PD: 65.0 ± 11.1 HC: 67.1 ± 7.4	PD: 28.6% HC: 45.3%	PD: 6.0 ± 4.6	PD: 2.2 ± 0.7	PD: 26.3 ± 13.8	PD: 27.6 ± 3.0	NR
Zhao et al. 2019 [50]	NR N Y	1500* g* for 20 min with thrombin to deplete the coagulation factors followed by ExoQuick (Systems Biosciences) treatment to the supernatant	L1CAM (clone 5G3)	TEM	Glycine–hydrochloride and DBS-2 with BSA and 0.10% Tween 20 and incubation for 10 min at 37 °C	Frozen at −80 °C after lysis	39 Early: 22 Advanced: 17	0	0	0	0	0	40	PD: 67.5 ± 6.9 Early: 65.2 ± 11.2 Advanced: 67.5 ± 6.8 HC: 66.6 ± 8.8	PD: 41.0% HC: 57.5%	PD: 5.0 ± 3.2 Early: 3.9 ± 2.5 Advanced: 6.4 ± 3.6	NR	PD: 48.6 ± 21.0 Early: 37.8 ± 15.2 Advanced: 62.6 ± 19.3	NR	NR
Niu et al. 2020 [25]	NR N N	2000* g* for 15 min to remove cell debris followed by 12,000* g* for 30 min to remove larger EVs/particles followed by direct IP	L1CAM (clone UJ127)	TEM TRPS WB for CD63 and L1CAM	1% Triton X-100	N	53 Early: 36 Advanced: 17	0	0	0	0	20	21	PD: 65.0 ± 5.3 Early: 64.2 ± 4.9 Advanced: 66.5 ± 5.9 RBD: 63.2 ± 6.0 HC: 64.0 ± 5.4	PD: 53.0% Early: 50.0% Advanced: 59.0% RBD: 40.0% HC: 48.0%	NR	PD: 2.0 ± 0.5 Early: 1.5 ± 0.5 Advanced: 3.0 ± 0.5 RBD: NR	PD: 22.3 ± 10.3 Early: 18.4 ± 7.5 Advanced: 29.7 ± 11.1 RBD: NR	PD: 27.6 ± 2.6 Early: 28.2 ± 1.6 Advanced: 26.5 ± 3.6 RBD: NR	PD: 23.6 ± 3.6 Early: 24.1 ± 3.3 Advanced: 22.5 ± 4.1 RBD: NR
Zou et al. 2020 [52]	EDTA Y (1600 × g for 20 min at NR °C) N	1000* g* for 20 min to remove cells followed by 1600* g* for 20 min to deplete platelets and samples were stored at − 80 °C. Thawed samples were centrifuged for 2,000* g* for 15 min followed by 12,000* g* for 30 min to remove larger EVs/particles followed by direct IP	L1CAM (clone UJ127)	TEM NTA WB for CD9, CD63 and TSG101	1% Triton X-100	N	93 Early: 51 Advanced: 42	0	0	0	0	0	85	PD: 66.9 ± 9.5 Early: 64.7 ± 10.6 Advanced: 67.5 ± 8.1 HC: 66.2 ± 10.3	PD: 43.0% Early: 43.1% Advanced: 42.9% HC: 43.5%	PD: 4.3 ± 2.5 Early: 2.0 ± 3.4 Advanced: 5.3 ± 3.1	PD: 2.8 ± 0.5 Early: 1.5 ± 0.5 Advanced: 3.0 ± 0.5	PD: 28.7 ± 16.0 Early: 22.0 ± 18.5 Advanced: 31.4 ± 15.3	PD: 24.3 ± 2.9 Early: 28.7 ± 2.6 Advanced: 21.9 ± 2.6	NR
Yu et al. 2020 [49]	NR N N	2000* g* for 15 min to remove cell debris followed by 12,000* g* for 30 min to remove larger EVs/particles followed by direct IP	L1CAM (clone UJ127) for nEVs CNPase (clone mABcam 44,289) for oEVs	NTA TEM WB for Alix, CD9 and CNPase	1% Triton X-100	N	34	32	0	0	0	0	31	PD: 63.6 ± 8.0 MSA: 63.0 ± 6.9 HC: 64.3 ± 7.5	PD: 41.2% MSA: 40.6% HC: 51.6%	PD: 4.0 ± 2.2 MSA: 4.0 ± 2.8	NR	PD: 21.4 ± 10.6 MSA (UMSARS): 24.1 ± 10.6	NR	NR
Yan et al. 2022 [48]	NR N Y	ExoQuick (Systems Biosciences)	L1CAM (clone UJ127)	NTA TEM WB for Alix, Flotillin-1 and CD63	RIPA	Frozen at −20 °C after lysis	44 Early: 28 Advanced: 16	0	0	0	0	101	48	PD: 64.2 ± 9.6 Early: 63.2 ± 9.8 Advanced: 65.9 ± 9.2 RBD: 61.9 ± 7.9 HC: 61.5 ± 7.1	PD: 56.8% Early: 53.6% Advanced: 62.5% RBD: 56.4% HC: 54.2%	PD: 3.7 ± 3.8 Early: 2.5 ± 3.0 Advanced: 5.7 ± 4.1 RBD: NR	PD: 2.1 ± 1.0 Early: 1.6 ± 0.5 Advanced: 3.1 ± 0.8 RBD: NR	PD: 32.0 ± 19.7 Early: 21.8 ± 10.7 Advanced: 49.7 ± 19.5 RBD: NR	PD: 23.7 ± 6.2 Early: 25.3 ± 4.8 Advanced: 21.1 ± 7.4 RBD: 21.8 ± 5.9	PD: 19.2 ± 7.1 Early: 21.0 ± 5.8 Advanced: 15.9 ± 8.2 RBD: 16.6 ± 6.4
Jiao et al. 2023 [18]	NR N N	2000* g* for 15 min to remove cell debris followed by 12,000* g* for 30 min to remove larger EVs/particles followed by direct IP	L1CAM (clone UJ127)	TEM TRPS WB for Alix and L1CAM	1% Triton X-100	N	Early: 50	0	0	0	0	0	50	PD: 64.3 ± 5.6 HC: 64.0 ± 5.8	PD: 56.0% HC: 50.0%	PD: 2.3 ± 1.3	PD: 1.6 ± 0.4	PD: 21.9 ± 8.6	PD: 28.1 ± 1.6	PD: 24.3 ± 3.0
Wang et al. 2023 [46]	EDTA N N	3200* g* for 15 min to remove cell debris and stored at −80 °C. Thawed samples were centrifuged for 2000* g* for 15 min followed by 12,000* g* for 30 min to remove larger EVs/particles followed by direct IP	L1CAM (clone UJ127) for nEVs GLT-1 (clone ABED-10) for aEVs	FC NTA TEM WB for Alix and CD9	NA	N	106	47	0	0	0	0	103	PD: 60.9 ± 13.9 MSA: 61.2 ± 12.3 HC: 56.5 ± 12.5	PD: 44.3% MSA: 46.8% HC: 43.7%	PD: 6.4 ± 4.4 MSA: 3.2 ± 1.6	NR	NR	NR	NR
Chen et al. 2023 [5]	NR N N	2000* g* for 20 min followed by 10,000* g* for 20 min to remove larger EVs/particles followed by Total Plasma Exosome Isolation Kit (ThermoFisher Scientific) followed by IP	L1CAM (clone 5G3)	TEM WB for CD63	0.2% Triton x-100	Frozen at −80 °C after lysis	43 Early: 16 Advanced: 27	0	0	0	0	0	34	PD: 66.6 ± 9.6 Early: 64.5 ± 10.8 Advanced: 67.9 ± 8.8 HC: 63.7 ± 10.0	PD: 39.5% Early: 56.2% Advanced: 29.6% HC: 50.0%	PD: 4.4 ± 3.5 Early: 2.3 ± 2.5 Advanced: 5.6 ± 3.8	PD: 2.7 ± 1.2 Early: 1.4 ± 0.5 Advanced: 3.5 ± 0.8	NR	NR	NR
*Serum*																				
Si et al. 2019 [39]	NA N NA	3000* g* for 15 min at 4 °C to remove cells and debris followed by ExoQuick (Systems Biosciences)	L1CAM (clone UJ127)	EM WB for CD9	1% Triton X-100	N	38	0	0	0	0	0	18	PD: 62.4 ± 9.7 TD: 62.7 ± 10.6 NTD: 62.1 ± 10.6 HC: 62.7 ± 2.3	PD: 50.0% TD: 45.4% NTD: 50.0% HC: 55.5%	PD: 2.3 ± 1.8 TD: 1.6 ± 1.2 NTD: 3.0 ± 2.5	PD: 1.7 ± 0.6 TD: 1.6 ± 0.6 NTD: 1.7 ± 0.5	PD: 18.6 ± 10.2 TD: 18.3 ± 9.4 NTD: 18.9 ± 10.9	NR	NR
Jiang et al. 2020 [17]	NA N NA	300* g* for 10 min, 2000* g* for 20 min and 10,000* g* to for 30 min to remove cellular debris, protein aggregates and fatty material followed by direct IP	L1CAM (clone UJ127)	NTA SEM WB for syntenin-1 and TSG101	1% Triton X-100	N	275 PDD: 45	14	21	35	45	65	144	PD: 68.9 ± 7.1 MSA: 68.1 ± 10.8 DLB: 68.5 ± 4.9 PSP: 68.0 ± 7.5 CBS: 61.1 + 7.2 RBD: 64.2 ± 8.3 HC: 68.1 ± 10.8	PD: 33.8% MSA: 40.0% DLB: 71.4% PSP: 48.6% CBS: 40.0% RBD: 4.6% HC: 34.7%	PD: 7.5 ± 7.0 MSA: 4.9 ± 2.6 DLB: 3.4 ± 3.0 PSP: 2.8 ± 1.8 CBS: 1.9 ± 1.3 RBD: NR	NR	PD: 32.2 ± NR MSA: 27.7 ± NR DLB: 20.9 ± NR PSP: 24.5 ± NR CBS: 22.5 ± NR RBD: 5.1 ± NR	NR	PD: 22.7 ± NR MSA: 16.9 ± NR DLB: 16.3 ± NR PSP: 21.4 ± NR CBS: 22.3 ± NR RBD: 25.5 ± NR
Agliardi et al. 2021 [1]	NA N NA	ExoQuick (Systems Biosciences)	L1CAM (clone 5G3)	Exo-Check Antibody Array NTA TEM WB for CD63, CD81, CD9, TSG101 and LCAM-1	M-PER	Frozen at −80 °C after lysis	32	0	0	0	0	0	40	PD: 69.5 ± 8.6 HC: 57.4 ± 7.6	PD: 34.4% HC: 47.5%	PD: 6.3 ± 3.6	PD: 2.0 ± NR	PD: 28.5 ± 13.2	NR	PD: 24.2 ± 2.5
Jiang et al. 2021 [16]	NA N NA	300* g* for 10 min, 2000* g* for 20 min and 10,000* g* for 30 min to remove cellular debris, protein aggregates and fatty material followed by direct IP	L1CAM (clone UJ127)	NTA SEM WB for syntenin-1 and TSG101	1% Triton X-100	N	290	50	0	116	88	0	191	PD: 65.1 ± 7.8 MSA: 67.1 ± 10.0 PSP: 69.5 ± 2.2 CBS: 64.6 ± 7.2 HC: 64.4 ± 6.8	PD: 34.8% MSA: 30.0% PSP: 37.9% CBS: 53.4% HC: 41.9%	PD: 7.4 ± 3.1 MSA: 5.2 ± 2.7 PSP: 3.5 ± 2.2 CBS: 3.3 ± 2.0	NR	PD: 25.9 ± NR MSA: 27.7 ± NR PSP: 31.0 ± NR CBS: 36.1 ± NR	NR	PD: 26.8 ± NR MSA: 26.0 ± NR PSP: 22.0 ± NR CBS: 20.9 ± NR
Dutta et al. 2021 [11]	NA N Y (few samples were obtained as plasma and treated with thrombin followed by high-speed centrifugation)	2000* g* for 10 min at 4 °C to remove cellular debris followed by ExoQuick (Systems Biosciences)	L1CAM (clone 5G3) for nEVs MOG (clone D-2) for oEVs	FC TEM TRPS WB for Alix, CD9, and CD81	RIPA	Frozen at −80 °C after lysis	104	80	0	0	0	0	101	PD: 66.8 ± 9.3 MSA: 62.8 ± 8.1 HC: 64.9 ± 10.5	PD: 55.5% MSA: 51.2% HC: 55.4%	PD: 7.0 ± 4.4 MSA: 5.0 ± 2.8	PD: 2.4 ± 0.9 MSA: 3.8 ± 1.9	PD: 20.7 ± 14.3 MSA: NR	PD: 27.0 ± 4.2 MSA 27.2 ± 5.5	NR
Meloni et al. 2023 [24]	NA N NA	4000* g* for 20 min at 4 °C followed by ExoQuick (Systems Biosciences)	L1CAM (clone 5G3)	Exo-Check Antibody Array NTA TEM WB for CD63, CD81, CD9, TSG101 and LCAM-1	M-PER	Frozen at −80 °C after lysis	70	0	0	21	19	0	0	PD: 69.5 ± 7.5 PSP: 72.8 ± 8.5 CBS: 71.9 ± 8.0	PD: 44.2% PSP: 47.6% CBS: 57.9%	PD: 7.3 ± 5.6 PSP: 4.0 ± 1.6 CBS: 4.4 ± 3.1	PD: 2.1 ± 0.6 PSP: NR CBS: NR	PD: 33.0 ± 14.4 PSP: NR CBS: NR	NR	PD: 25.1 ± 2.7 PSP: 17.8 ± 5.1 CBS: 17.0 ± 8.1
Taha et al. 2023 [43]	NA N Y (few samples were obtained as plasma and treated with thrombin followed by high-speed centrifugation)	2000* g* for 10 min at 4 °C to remove cellular debris followed by ExoQuick (Systems Biosciences)	L1CAM (clone 5G3) for nEVs MOG (clone D-2) for oEVs	FC TEM TRPS WB for Alix, CD9, and CD81	RIPA	Frozen at −80 °C after lysis	46	30	0	0	0	0	32	PD: 66.8 ± 11.6 MSA: 62.7 ± 8.2	PD: 46.8% MSA: 56.7%	PD: 8.1 ± 5.0 MSA: 62.7 ± 8.2	PD: 2.5 ± 1.0 MSA: 3.8 ± 1.0	PD: 25.1 ± 15.6	PD: 26.3 ± 6.4 MSA: 26.5 ± 9.3	NR
Sharafeldin et al. 2023 [35]	NA N NA	On-Chip Immunocapture	L1CAM (clone UJ127)	DLS FM NTA WB for syntenin-1 and TSG101	1% Triton X-100	N	20	0	0	0	0	23	29	NR	NR	NR	NR	NR	NR	NR

*aEVs* astrocyte extracellular vesicles; *Alix* ALG-2-interacting protein X; *CBS* corticobasal syndrome; *CFM* confocal fluorescence microscopy; *CNPase* 2′,3′-cyclic-nucleotide 3′-phosphodiesterase; *DLB* dementia with Lewy body; *DLS* dynamic light scattering; *ECLIA* electrochemilumiscence ELISA; *EDTA* ethylenediaminetetraacetic acid; *ELISA* enzyme-linked immunosorbent assay; *EM* electron microscopy; *EV*– extracellular vesicle; *FC* flow-cytometry; *FM* fluorescence microscopy; *GLT-1* glutamate transporter 1; *HC* healthy control; *HY* Hoehn and Yahr disease stage scale45; *IP* immunoprecipitation; *L1CAM* L1 cell adhesion molecule; *MCI* mild cognitive impairment; *MMSE* mini-mental state examination; *MoCA* Montreal cognitive assessment; *MOG* myelin oligodendrocyte glycoprotein; *M-PER* mammalian protein extraction reagent; *MPs-CILA* paramagnetic particle-based chemiluminescence immunoassay; *MSA* multiple system atrophy; *NA* not applicable; *NC* non-cognitively impaired; *nEVs* neuronal extracellular vesicles; *NR* not reported; *NTA* nanoparticle tracking analysis; *NTD* non tremor-dominant; *oEVs* oligodendrocyte extracellular vesicles; *PD* Parkinson's disease; *PDD* PD with dementia; *pS129-α-syn* phosphorylated α-syn at Ser 129; *PSP* progressive supranuclear palsy; *RIPA* radioimmunoprecipitation assay; *SEM* scanning EM; *TD* tremor-dominant; *TEM* transmission EM; *TRPS* tunable resistive pulse sensing; *TSG101* tumor susceptibility gene 101 protein; *UMSARS* unified multiple system atrophy rating scale48; *UPDRSIII* Unified Parkinson's disease rating scale.49; *WB* Western blot

The studies included in the analysis were generally of high quality as indicated in Table S3. However, there was a lack of clear reporting on the sampling method, which made the assessment of the risk of bias in patient selection unclear. One study excluded participants from their analysis and was deemed to have a high risk of bias [48]. The measurement of biomarkers in speculative nEVs and oEVs was considered to have a low risk of bias in the index test domain, as it is an objective measure unaffected by prior knowledge of the clinical status. While the majority of the articles (66.7%) had a low risk of bias in the reference standard domain, four studies using an in-house test [12, 35, 38, 43, 49] and one using WBs [48] were identified as having a high risk of bias. In terms of the Flow and Timing domain, all studies were deemed to have a low risk of bias as the time interval from clinical diagnosis to biomarker measurement could be reliably estimated.

As previously indicated [40–42], several pre-analytical elements can have a substantial effect on the purity, content, dimensions, and amount of EVs. Such elements involve the selection of anticoagulation molecules mixed with plasma, EV isolation methodology, the centrifugation procedure, the transportation characteristics, the frequency of freezing and thawing cycles, the storage parameters, the temperature, and the type of tube used for collection. Regrettably, these aspects are not universally standardized across biobanks or methods of clinical lab blood collection. Moreover, the use of the anti-L1 cell adhesion molecule (L1CAM) antibody clone UJ127 has initiated doubts regarding its possible cross-reactivity with α-syn antibodies [26].

To tackle these concerns, we performed subgroup analyses based on the medium (either plasma or serum) and the type of antibody clone (e.g., L1CAM clone UJ127 or 5G3) for the analyses for patients with PD vs HCs. We did not perform such analyses for other diseases due to the small number of studies included.

Descriptive statistics of the meta diagnostic analysis including the sensitivity, specificity, FPR, diagnostic odds ratio (DOR), positive likelihood ratio (posLR), and negative likelihood ratio (negLR) for each included analysis are summarized in Table 2.

**Table 2:** Descriptive statistics of the diagnostic metrics of studies included in the meta-analysis

Study	Biomarkers	Sensitivity (95% CI)	Specificity (95% CI)	FPR (95% CI)	DOR (95% CI)	posLR (95% CI)	negLR (95% CI)
*PD vs control*							
Shi et al. 2014 [38]	nEVs α-syn	0.701 (0.659–0.740)	0.529 (0.484–0.573)	0.471 (0.427–0.516)	2.64 (2.02–3.44)	1.49 (1.33–1.66)	0.565 (0.481–0.663)
Shi et al. 2014 [38]	nEVs α-syn/total plasma α-syn	0.712 (0.670–0.750)	0.500 (0.456–0.544)	0.500 (0.456–0.544)	2.47 (1.89–3.22)	1.42 (1.28–1.58)	0.577 (0.488–0.681)
Shi et al. 2016 [37]	nEVs tau	0.701 (0.659–0.740)	0.529 (0.484–0.573)	0.350 (0.287–0.419)	2.55 (1.70–3.83)	1.65 (1.32–2.07)	0.648 (0.535–0.787)
Zhao et al. 2019 [50]	nEVs DJ-1/total plasma DJ-1	0.595 (0.485–0.696)	0.823 (0.724–0.891)	0.177 (0.109–0.276)	6.82 (3.28–14.17)	3.36 (2.02–5.58)	0.492 (0.370–0.655)
Zhao et al. 2019 [50]	nEVs α-syn + DJ-1	0.823 (0.724–0.891)	0.519 (0.411–0.626)	0.481 (0.374–0.589)	5.01 (2.42–10.36)	1.71 (1.33–2.20)	0.341 (0.203–0.575)
Niu et al. 2020 [25]	nEVs α-syn	0.973 (0.907–0.993)	0.541 (0.428–0.649)	0.459 (0.351–0.572)	42.35 (9.67–185.60)	2.12 (1.65–2.72)	0.050 (0.013–0.199)
Niu et al. 2020 (early-stage PD vs HC) [25]	nEVs α-syn	0.095 (0.047–0.183)	0.568 (0.454–0.674)	0.432 (0.326–0.546)	0.14 (0.06–0.34)	0.22 (0.10–0.46)	1.595 (1.290–1.972)
Zou et al. 2020 [52]	nEVs α-syn, Linc-POU3F3, and plasma GCase activity	0.708 (0.637–0.770)	0.831 (0.770–0.879)	0.169 (0.121–0.230)	11.95 (7.19–19.87)	4.20 (2.99–5.90)	0.351 (0.277–0.446)
Si et al. 2019 [39]	nEVs α-syn	0.661 (0.530–0.771)	0.714 (0.585–0.816)	0.286 (0.184–0.415)	4.87 (2.19–10.85)	2.31 (1.47–3.64)	0.475 (0.318–0.710)
Jiang et al. 2020 [17]	nEVs α-syn	0.850 (0.812–0.881)	0.740 (0.696–0.780)	0.260 (0.220–0.304)	16.07 (11.38–22.71)	3.27 (2.77–3.86)	0.203 (0.161–0.257)
Agliardi et al. 2021 [1]	nEVs α-syn/STX-1A	0.861 (0.763–0.923)	0.819 (0.715–0.891)	0.181 (0.109–0.285)	28.14 (11.46–69.08)	4.77 (2.89–7.87)	0.169 (0.094–0.304)
Agliardi et al. 2021 [1]	nEVs α-syn/VAMP-2	0.750 (0.639–0.836)	0.931 (0.848–0.970)	0.069 (0.030–0.152)	40.20 (14.02–115.30)	10.80 (4.59–25.42)	0.269 (0.179–0.403)
Jiang et al. 2021 [16]	nEVs α-syn	0.819 (0.782–0.851)	0.740 (0.699–0.777)	0.260 (0.223–0.301)	12.90 (9.47–17.57)	3.15 (2.70–3.69)	0.244 (0.201–0.298)
Jiang et al. 2021 [16]	nEVs α-syn/Clusterin	0.861 (0.827–0.889)	0.869 (0.836–0.896)	0.131 (0.104–0.164)	40.99 (28.32–59.34)	6.57 (5.21–8.30)	0.160 (0.128–0.201)
Dutta et al. 2021 [11]	nEVs α-syn, oEVs/nEVs α-syn, and CD81 concentration	0.712 (0.618–0.790)	0.625 (0.529–0.712)	0.375 (0.288–0.471)	4.11 (2.30–7.35)	1.90 (1.44–2.50)	0.462 (0.330–0.646)
Yan et al. 2022 [48]	EVs and nEVs α-syn	0.837 (0.748–0.899)	0.663 (0.562– 0.751)	0.337 (0.249–0.438)	10.1 (5.01–20.38)	2.48 (1.84– 3.35)	0.246 (0.151–0.400)
Taha et al. 2023 [43]	nEVs α-syn, oEVs/nEVs α-syn, oEVs pS129-α-syn, and CD81 concentration	0.718 (0.610–0.806)	0.897 (0.810–0.947)	0.103 (0.053–0.190)	22.27 (9.22–53.82)	7.00 (3.58–13.69)	0.314 (0.219–0.451)
Taha et al. 2023 [43]	nEVs α-syn, oEVs/nEVs α-syn, oEV tau, and CD81 concentration	0.419 (0.329–0.515)	0.848 (0.767–0.904)	0.152 (0.096–0.233)	4.01 (2.08–7.75)	2.75 (1.66–4.55)	0.685 (0.572–0.822)
Sharafeldin et al. 2023 [35]	nEVs α-syn	0.650 (0.495–0.779)	0.950 (0.835–0.986)	0.050 (0.0138–0.165)	35.3 (7.39–168.4)	13.0 (3.30–51.1)	0.368 (0.240–0.565)
Jiao et al. 2023 [18]	nEVs α-syn, plasma CCL2, and CXCL12	0.680 (0.583–0.763)	0.940 (0.875–0.972)	0.060 (0.0277–0.124)	33.3 (13.2–84.0)	11.3 (5.16–24.9)	0.340 (0.255–0.455)
Wang et al. 2023 [46]	GLT-1 + and SYN211 + EVs and GLT-1 + and MFJR14 + EVs	0.818 (0.760–0.865)	0.895 (0.846–0.929)	0.10 (0.070–0.15)	38.25 (21.75–62.27)	7.77 (5.21–6.10)	0.20 (0.15–0.27)
Chen et al. 2023 [5]	nEVs Transferrin R-Protein	0.857 (0.762–0.918)	0.701 (0.592–0.792)	0.30 (0.21–0.41)	14.09 (6.31–31.46)	2.87 (2.01–4.09)	0.36 (0.11–0.36)
*PD vs MSA*							
Yu et al. 2020 [49]	oEVs α-syn	0.621 (0.501–0.729)	0.818 (0.709–0.893)	0.182 (0.107–0.292)	7.38 (3.32–16.41)	3.42 (1.98 –5.89)	0.463 (0.333–0.643)
Yu et al. 2020 [49]	oEVs α-syn/total plasma α-syn	0.530 (0.412–0.646)	0.848 (0.743–0.916)	0.152 (0.0844–0.257)	6.32 (2.76–14.48)	3.50 (1.89–6.47)	0.554 (0.42098–0.729)
Jiang et al. 2020 [17]	nEVs α-syn + Clusterin	0.958 (0.929–0.976)	0.920 (0.883–0.946)	0.0796 (0.0536–0.117)	266.96 (130.21–547.34)	12.04 (8.13–17.84)	0.045 (0.026–0.079)
Jiang et al. 2021 [16]	nEVs α-syn	0.820 (0.728–0.886)	0.865 (0.779–0.921)	0.135 (0.0788–0.221)	29.28 (12.97–66.08)	6.08 (3.56 –10.39)	0.208 (0.132–0.326)
Jiang et al. 2021 [16]	nEVs α-syn/Clusterin	0.910 (0.833–0.954)	0.640 (0.537–0.732)	0.360 (0.268–0.463)	18.04 (7.74–42.01)	2.53 (1.90 –3.36)	0.140 (0.071–0.277)
Dutta et al. 2021 [11]	nEVs α-syn, oEVs/nEVs α-syn, and CD81 concentration	0.893 (0.819–0.939)	0.864 (0.785–0.917)	0.136 (0.0827–0.215)	53.17 (22.91–123.37)	6.57 (4.02–10.74)	0.124 (0.070–0.217)
Taha et al. 2023 [43]	nEVs α-syn, oEVs/nEVs α-syn, oEVs pS129-α-syn, and CD81 concentration	0.803 (0.700–0.877)	0.895 (0.806–0.946)	0.105 (0.0543–0.194)	34.57 (13.71–87.18)	7.63 (3.92–14.83)	0.221 (0.139–0.349)
Taha et al. 2023 [43]	nEVs α-syn, oEVs:nEVs α-syn, oEVs tau, and CD81 concentration	0.946 (0.879–0.977)	0.707 (0.607–0.790)	0.293 (0.210–0.393)	41.89 (15.30–114.65)	3.22 (2.34–4.44)	0.077 (0.032–0.182)
Wang et al. 2023 [46]	GLT-1 + SYN211 + EVs and GLT-1 + and MFJR14 + EVs	0.784 (0.713–0.842)	0.895 (0.837–0.935)	0.10 (0.065–0.16)	31.14 (16.33–59.37)	7.50 (4.68–12.01)	0.241 (0.177–0.327)
*PD vs PSP and CBS*							
Jiang et al. 2020 (FTD included) [17]	nEVs α-syn + Clusterin	0.918 (0.888–0.941)	0.958 (0.935–0.974)	0.042 (0.026–0.065)	258.31 (142.92–466.86)	22.09 (13.95–34.97)	0.086 (0.062–0.118)
Jiang et al. 2021 [16]	nEVs α-syn/Clusterin	0.999 (0.990–1.000)	0.948 (0.925–0.965)	0.052 (0.035–0.075)	18,209.24 (1105.43–299,953.43)	19.39 (13.29–28.30)	0.001 (0.000–0.017)
Meloni et al. 2023 [24]	nEVs α-syn/tau	0.941 (0.881–0.972)	0.671 (0.579–0.752)	0.329 (0.248–0.421)	32.82 (13.53–79.57)	2.86 (2.19–3.75)	0.087 (0.041–0.186)
*MSA vs control*							
Yu et al. 2020 [49]	oEVs α-syn	0.836 (0.727–0.907)	0.711 (0.590–0.808)	0.289 (0.192–0.410)	12.53 (5.33–29.44)	2.89 (1.94–4.31)	0.231 (0.130–0.410)
Yu et al. 2020 [49]	oEVs α-syn/total plasma α-syn	0.523 (0.403–0.641)	0.555 (0.433–0.670)	0.445 (0.330–0.567)	1.37 (0.68–2.74)	1.18 (0.82–1.68)	0.859 (0.613–1.204)
Dutta et al. 2021 [11]	nEVs α-syn, oEVs/nEVs α-syn, and CD81 concentration	0.956 (0.897–0.982)	0.838 (0.755–0.897)	0.162 (0.103–0.245)	112.27 (38.05–331.28)	5.91 (3.79–9.21)	0.053 (0.021–0.130)
Taha et al. 2023 [43]	nEVs α-syn, oEVs/nEVs α-syn, oEVs pS129-α-syn, and CD81 concentration	0.992 (0.928–0.999)	0.960 (0.880–0.988)	0.040 (0.012–0.120)	3025.00 (142.28–64,314.30)	25.00 (7.42–84.25)	0.008 (0.001–0.131)
Taha et al. 2023 [43]	nEVs α-syn, oEVs:nEVs α-syn, oEVs tau, and CD81 concentration	0.880 (0.800–0.931)	0.922 (0.851–0.961)	0.078 (0.039–0.149)	86.70 (32.97–228.04)	11.27 (5.65–22.49)	0.130 (0.075–0.224)
*RBD vs control*							
Jiang et al. 2020 [17]	nEVs α-syn	0.610 (0.530–0.684)	0.810 (0.742–0.865)	0.188 (0.133–0.258)	6.67 (4.00–11.48)	3.25 (2.27–4.64)	0.479 (0.386–0.595)
Yan et al. 2022 [48]	nEVs α-syn	0.936 (0.894–0.962)	0.717 (0.652–0.773)	0.282 (0.226–0.346)	38.33 (20.3–72.5)	3.32 (2.67–4.13)	0.087 (0.051–0.148)
Sharafeldin et al. 2023 [35]	nEVs α-syn	0.682 (0.511–0.814)	0.985 (0.870–0.998)	0.0151 (0.00158–0.129)	139.3 (7.76–2500.0)	45.0 (2.84–711.4)	0.323 (0.196–0.533)

*95% CI* 95% confidence interval; *CCL2* C–C motif chemokine ligand 2; *CBS* corticobasal syndrome; *CXCL12* C-X-C motif chemokine ligand 12; *DJ-1* protein deglycase DJ-1; *DOR* diagnostic odds ratio; *EV* extracellular vesicle; *FPR* false-positive rate; *FTD* frontotemporal dementia; *GCase* glucocerebrosidase; *HC* healthy control; *Linc-POU3F3* long intergenic noncoding RNA POU3F3; *MSA* multiple system atrophy; *negLR* negative likelihood ratio; *nEVs* neuronal extracellular vesicles; *oEVs* oligodendroglial extracellular vesicles; *PD* Parkinson's disease; *posLR* positive likelihood ratio; *PSP* progressive supranuclear palsy; *pS129-α-syn* phosphorylated Serine 129 α-synuclein; *RBD* REM behavior disorder; *α-synuclein* α-syn; *STX-1A* syntaxin-1A; *VAMP-2* vesicle associated membrane protein 2

### PD vs control

**Fig. 2:**
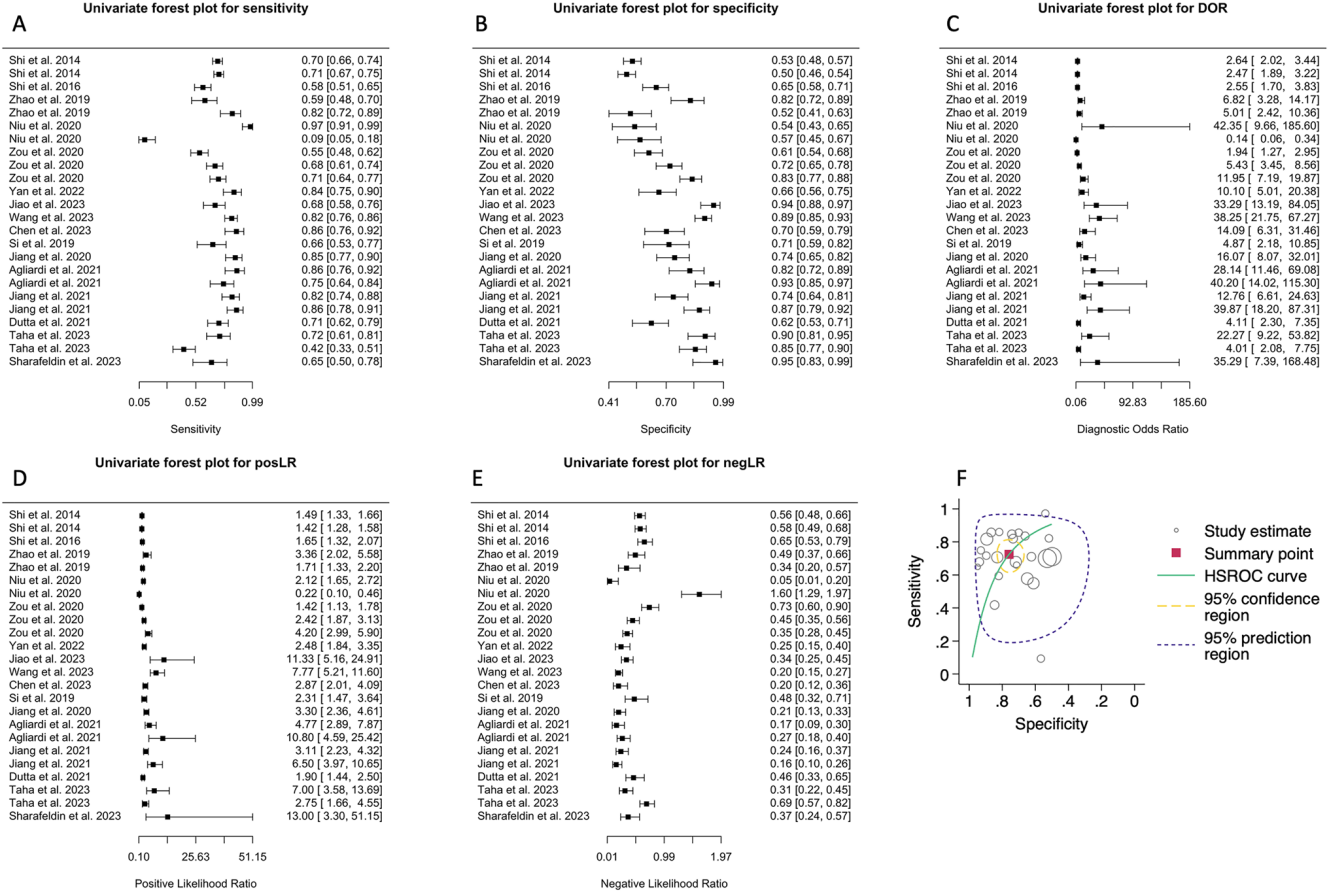
Diagnostic accuracy of biomarkers in speculative CNS-enriched EVs for the differential diagnosis of Parkinson’s disease (PD) from healthy controls (HCs). **A-E** Univariate Forest plots for sensitivity, specificity, diagnostic odds ratio (DOR), positive (posLR) and negative (negLR) likelihood ratios, respectively. **F** Summary receiver operating characteristics (SROC). The dotted circle shows the mean summary estimate of sensitivities and specificities using a bivariate model. The summary line is obtained from a hierarchical SROC model. *CNS* central nervous system; *EVs* extracellular vesicles

Sixteen studies attempted to differentiate patients with PD from HCs using biomarkers in speculative nEVs [1, 5, 11, 16–18, 25, 35, 37–39, 43, 48, 50, 52], oEVs [11, 43], and/or aEVs [46]. The AUC ranged between 0.610 and 0.915 with the highest AUC obtained in 2023 from Wang et al. [46], while the sensitivity (Fig. 2A) and specificity (Fig. 2B) ranged between 0.10–0.97 and 0.50–0.95, respectively. The chi-square (χ2) equality test revealed high heterogeneity for sensitivity (χ^2^ = 312.45, df = 23, *p* < 0.0001) and specificity (χ^2^ = 345.19, df = 223, *p* < 0.0001). Both crosshair and ROC ellipse plots confirmed the heterogeneity present (Fig. S1A, B). Univariate Forest plots of the DOR, posLR, and negLR for each individual analysis are shown in Fig. 2C–E.

Bivariate and HSROC models (Table 3), each demonstrated a fair discriminatory ability of the diagnostic test. These models independently suggested that measuring biomarkers in speculative CNS-enriched EVs achieved fair accuracy in distinguishing patients with PD from HCs.

**Table 3:** Meta-analysis of diagnostic accuracy for patients with Parkinson’s disease vs healthy controls summary statistics for the bivariate and hierarchal summary receiver operating characteristic (HSROC) models

Model	Variable	Coefficient estimate ± SE (95% CI)
Summary statistic	Sensitivity	0.725 ± 0.038 (0.644–0.793)
	Specificity	0.759 ± 0.031 (0.692–0.814)
	DOR	8.29 ± 2.15 (4.98–13.79)
	posLR	3.00 ± 0.423 (2.28–3.96)
	negLR	0.362 ± 0.053 (0.272–0.482)
	1/negLR	2.76 ± 0.403 (2.07–3.67)
Bivariate	Logit-transformed sensitivity	0.970 ± 0.191 (0.595–1.34)
	Logit-transformed sensitivity variance	1.14 ± 0.170 (0.812–1.48)
	Logit-transformed specificity	0.813 ± 0.272 (0.421–1.57)
	Logit-transformed specificity variance	0.618 ± 0.207 (0.320–1.19)
	Correlation between sensitivity and specificity	0.035 ± 0.222 (-0.381–0.439)
	AUC (partial AUC)	0.800 (0.692)
HSROC	Lambda (Λ)	2.13 ± 0.261 (1.62–2.64)
	Theta (Θ)	-0.160 ± 0.172 (-0.498–0.178)
	Beta (β)	-0.137 ± 0.237 (-0.601–0.327)
	Variance Λ	1.47 ± 0.468 (0.785–2.74)
	Variance Θ	0.342 ± 0.113 (0.179–0.655)

Heterogeneity (*I*^2^) values showed significant variations depending on the approach utilized. Zhou and Dendukuri [51] reported a value of 35.1%, while Holling's sample size unadjusted [33] had values ranging from 87.7% to 93.5%, and the adjusted values ranging between 7.3% and 9.2%. However, all approaches generally indicated substantial heterogeneity across the studies, suggesting that the variability in the results cannot be attributed solely to random chance but rather to differences between the studies themselves, supporting the crosshair and ROC ellipse plots. It is also in agreement with the fact that studies measuring biomarkers in speculative CNS-enriched EVs generally suffer from failure of independent validation, which could be due to methodological and expertise heterogeneities. Though, as mentioned in the introduction, failure of independent validation and replication is often observed. This is the case even when the same methodology is employed.

The HSROC for this model is provided in Fig. 2F. The model suggested that measurement of biomarkers in speculative CNS-enriched EVs for distinguishing patients with PD from HCs may not be promising. The summary line shows an inverse relationship between sensitivity and specificity, indicative of a threshold effect, with the line being far away from the upper left corner. Moreover, while some studies achieved high sensitivity and specificity, the combined mean indicated that this test only achieves a fair distinguishing ability.

Importantly, few studies subdivided patients with PD to early vs advanced stages [5, 18, 25, 48, 50, 52] or only included patients with early-stage PD [39] using the Hoehn and Yahr scale: early-PD: 1–2 and late-PD: 3–5 [5, 50, 52], early-PD: ≤ 2 [25, 48], ≤ 2 with disease duration < 5 years [18] or 1–2.5 and drug-naïve [39]. Two of these studies [18, 25] attempted to differentiate patients with early-stage PD from HCs from similar research groups. Unfortunately, one of them [25] had the lowest sensitivity and specificity. This suggested that biomarkers in speculative CNS-enriched EVs may not be a good way to discriminate early-stage patients with PD from HCs despite it being the most clinically desired outcome of the test.

**Fig. 3:**
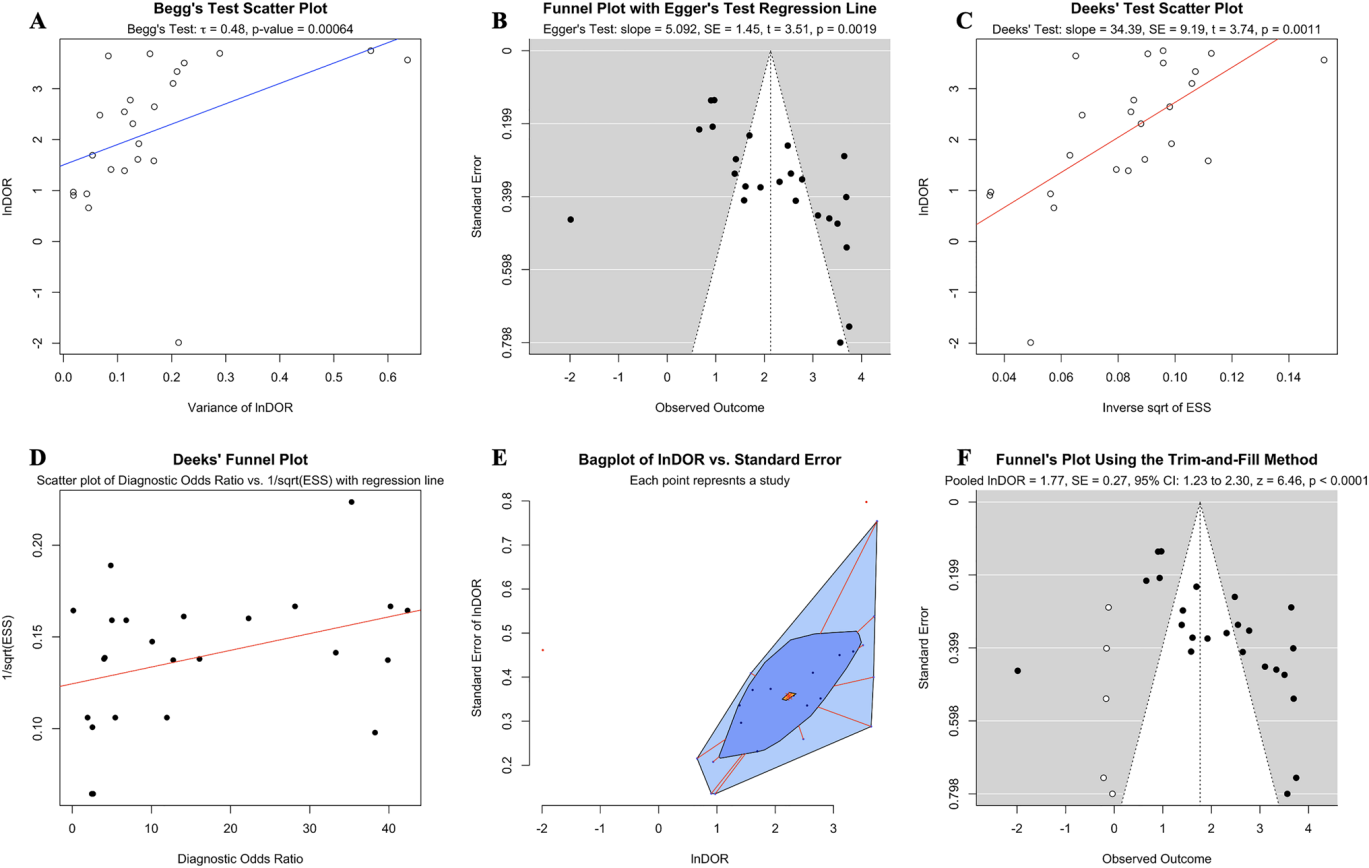
Publication bias was assessed using **A** Begg’s correlation, **B** Egger’s regression, **C** Deek’s regression, **D** Deek’s funnel plot, **E** A bagplot and **F** Funnel plot after application of the trim-and-fill method for biomarkers in speculative CNS-enriched EVs for the differential diagnosis of Parkinson’s disease from healthy controls. Collectively, they suggested a substantial presence of publication bias. The trim-and-fill method estimated five missing studies (white circles) on the left side of the figure with either small or null diagnostic accuracy. *CNS* central nervous system; *EVs* extracellular vesicles

All statistical tests conducted to assess publication bias in our analysis consistently indicated the presence of such bias. Begg’s correlation test revealed a significant positive correlation between lnDOR and its variance (tau = 0.48, *p* value = 0.0016; Fig. 3A), implying that larger effect sizes were associated with greater variances. Similarly, Egger’s regression test showed a significant positive relationship between the lnDOR and the standard error of the lnDOR (slope = 5.092, SE = 1.45 *t* = 3.51, *p* = 0.0019; Fig. 3B), suggesting that smaller studies, which tend to have larger standard errors, were reporting larger effect sizes than what would be expected if there was no bias. Finally, Deek’s regression test also indicated potential publication bias, with a significant positive slope (slope = 34.39, SE = 9.19, *t* = 3.74, *p* = 0.0011; Fig. 3C) showing that studies with smaller effective sample sizes were associated with larger effect sizes. Further examination of publication bias using Deek’s funnel plot (Fig. 3D) and a bivariate bagplot (Fig. 3E) also suggested the presence of publication bias.

Importantly, Duval and Tweedie's trim-and-fill method [36], a non-parametric method of adjusting for publication bias, estimated that there were approximately 5 studies missing from our meta-analysis due to publication bias. These missing studies are hypothesized to be on the left side of the funnel plot (see white circles in Fig. 3F), indicating smaller studies with lower DORs, and therefore may explain why they were not published. When these missing studies were imputed and included in a random-effects model, the adjusted diagnostic odds ratio became 1.77 (SE = 0.27, 95% CI: 1.23–2.30, *z* = 6.46, *p* < 0.0001). This suggested that when adjusting for potential publication bias, the diagnostic effect for using biomarkers in speculative CNS-enriched EVs for patients with PD vs HCs is much smaller than what is reported in the literature.

Collectively, the hierarchical bivariate model revealed moderate diagnostic accuracy of patients with PD from HCs using biomarkers in speculative CNS-enriched EVs, but with high heterogeneity and unreliability. Publication bias analyses showed that smaller studies with non-significant or low effects size results have been less likely to be published. Unsurprisingly, this is to be expected as alluded to previously [40, 41], there has been consistent failure of independent validation across studies using speculative CNS-enriched EVs, likely due to EVs being very sensitive to various pre-analytical factors [40], high complexity of methodologies used to isolate speculative CNS-enriched EVs as well as user differences in handling, among others. Even though measuring biomarkers in speculative CNS-enriched EVs for patients with PD vs HCs has been popular since 2014, only few studies currently exist, further indicating that studies with null results might not have been published. When the trim-and-fill method was used to account for the estimated five missing studies, the diagnostic effect for patients with PD vs HCs decreased substantially.

### PD vs control: sub-analysis by media, antibody clone, and quantification methodology

As described above, several pre-analytical factors may affect the EV signature obtained from plasma or serum. Recent studies suggested that plasma provides superior accuracy and reliability in comparison to serum for EV biomarker analysis [40], while the anti-L1CAM antibody clone UJ127 has been reported to cross-react with α-syn proteoforms [26].

**Fig. 4:**
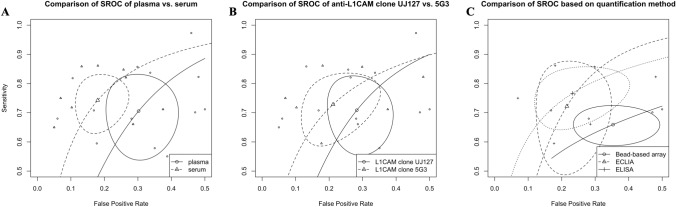
Summary receiver operating characteristics (SROC) comparing isolation of speculative CNS-enriched EVs using **A** plasma vs serum, **B** the anti-L1CAM antibody clone UJ127 vs 5G3 and **C** quantification methodology. *CNS* central nervous system; *ECLIA* electrochemilumiscence ELISA; *ELISA* enzyme-linked immunosorbent assay; *EVs* extracellular vesicles; *L1CAM* L1 cell adhesion molecule

In the present meta-analysis, we observed distinct differences between studies using plasma and serum. The plasma model (Table S4) yielded an overall lower diagnostic accuracy in comparison to the serum model (Table S5). Comparison of the hierarchical bivariate HSROC (Fig. 4A) obtained from studies using plasma [5, 18, 25, 37, 38, 46, 48, 50, 52] or serum [1, 11, 16, 17, 35, 39, 43] also suggested that the studies using serum had, on average, slightly better accuracy, though there was a decent overlap in the confidence intervals of both models.

It should also be noted that three [16, 17, 35] and two [11, 43] studies using serum, respectively, originated from the same research group while the majority of studies using plasma originated from unique research groups, suggesting a potential overlap in methodologies in studies using serum. Another potential explanation for the discrepancy in accuracy between plasma and serum studies is the way coagulation factors are handled. Many of the studies using plasma did not treat it with thrombin followed by a high-speed centrifugation to remove these factors despite using ExoQuick, a polymer-based precipitation technique, for EV isolation. ExoQuick's guidelines recommend removing the coagulation factors to prevent the precipitation of an insoluble  fibrin pellet after addition of ExoQuick and subsequent centrifugation, which can potentially skew the measurements. Moreover, the current scientific literature lacks details on how these coagulation factors or the presence of a fibrin pellet might impact the quantification of biomarkers within EVs. As such, these differences in the number of studies and potential methodological biases do not definitively establish one medium as superior over the other. Further independent studies focusing on these issues are needed to draw more conclusive comparisons.

Comparisons of studies using the anti-L1CAM antibody clone UJ127 (Table S6) vs 5G3 (Table S7) showed that studies using the 5G3 clone obtained a slightly higher accuracy though significant overlap was observed (Fig. 4B). Moreover, the studies included quantified α-syn using bead-based techniques (e.g., Simoa and Luminex), electrochemilumiscence ELISA (ECLIA) or ELISA, and as such, we compared the diagnostic accuracy of these methodologies. The results (Fig. 4C, Table S8) showed that ECLIA and ELISA obtained similar accuracies while bead-based methods achieved the lowest accuracy. We did not perform additional sub-analyses due to the small number of studies.

### PD vs HCs: speculative CNS-enriched EVs vs general EVs

As EVs are speculated to communicate cell-state-specific messages from the CNS to the peripheral circulation, measurement of biomarkers in speculative CNS-enriched EVs to distinguish patients with PD from HCs has been popular [13]. Speculative CNS-enriched EVs are often captured through direct immunoprecipitation or as a part of a two-step procedure where EVs are first isolated using a polymer-based precipitation technique (e.g., ExoQuick) or ultracentrifugation and nEVs, oEVs, or aEVs are immunoprecipitated using beads coupled to the chosen antibodies. Herein, we compared the diagnostic accuracy of biomarkers in general EVs [42] vs speculative CNS-enriched EVs.

Comparison of the bivariate and HSROC model statistics revealed that biomarkers in general EVs [42] have a higher diagnostic accuracy vs speculative CNS-enriched EVs (Table 4). Both methodologies showed evidence of publication bias, but the trim-and-fill method identified fewer missing studies in general EV biomarkers (2 out of 21) compared to speculative CNS-enriched EV biomarkers (5 out of 16), suggesting less publication bias in the former. We observed that only a single study [48] used biomarkers in general EVs and speculative CNS-enriched EVs for distinguishing between patients with PD and HCs. The rationale for the omission of such biomarkers in general EVs for diagnosis before transitioning to speculative CNS-enriched EVs remains unclear. It's important to highlight that isolation of speculative CNS-enriched EVs is notably more complex, time-consuming, and labor-intensive than general EVs.

**Table 4:** Comparison between general EVs [42] vs speculative CNS-enriched EVs for diagnosing patients with Parkinson’s disease (PD) from healthy controls using a bivariate and hierarchal summary receiver operating characteristics (HSROC) model. Reproduced from Taha et al. [42]

EV source	Mean sensitivity (95% CI)	Mean specificity (95% CI)	Pooled AUC (partial AUC))	Mean DOR ± SE (95% CI)
General EVs [42]	84.4% (77.7–90.7%)	79.1% (72.5–84.0%)	0.852 (0.672)	21.6 ± 1.3 (12.0–38.9)
CNS-enriched EVs	72.5% (64.4–79.3%)	75.9% (69.2–81.4%)	0.800 (0.692)	8.3 ± 2.1 (5.0–13.8)

The sensitivity, specificity, pooled area under the curve (AUC) and partial AUC, focusing on a specific range of false-positive rates (FPR), are obtained using the bivariate model. The diagnostic odds ratio (DOR) is obtained from the HSROC model

*EV* extracellular vesicles; *CNS* central nervous system; *SE* standard error

### PD vs MSA

**Fig. 5:**
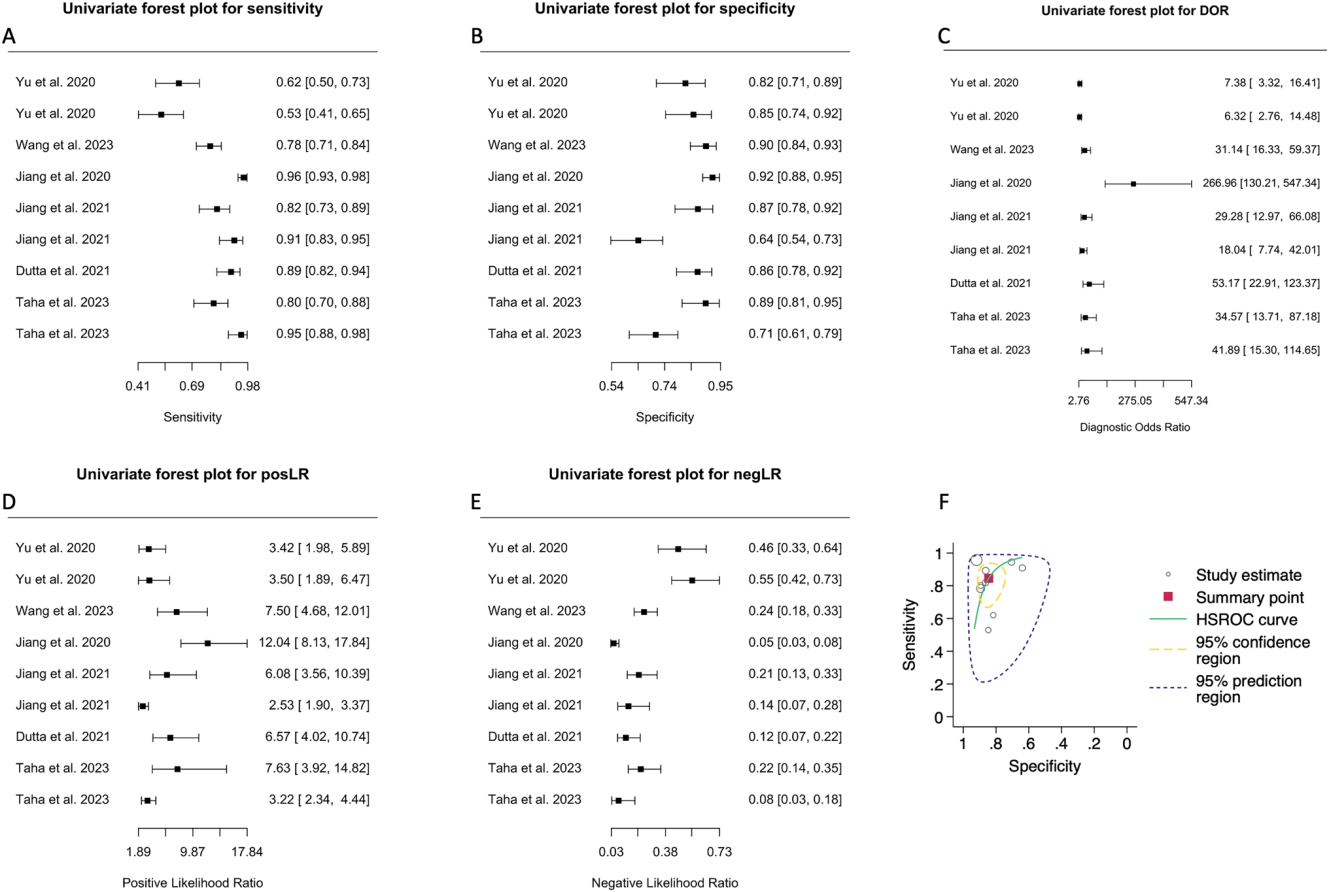
Diagnostic accuracy of biomarkers in speculative CNS-enriched EVs for the differential diagnosis of Parkinson’s disease (PD) from multiple system atrophy (MSA). **A**–**E** Univariate Forest plots for sensitivity, specificity, diagnostic odds ratio (DOR), positive (posLR) and negative (negLR) likelihood ratios, respectively. **F** Summary receiver operating characteristics (SROC). The dotted circle shows the mean summary estimate of sensitivities and specificities using a bivariate model. The summary line is obtained from a hierarchical SROC model. *CNS* central nervous system; *EVs* extracellular vesicles

Only six studies attempted to differentiate patients with PD from MSA [11, 16, 17, 43, 46, 49]. The AUC ranged between 0.709 and 0.980 while the sensitivity (Fig. 5A) and specificity (Fig. 5B) ranged between 0.53–0.96 and 0.64–0.92, respectively. Similarly, to the above, the chi-square (χ^2^) equality test revealed high heterogeneity for sensitivity (χ^2^ = 131.63, df = 7, *p* < 0.0001) and specificity (χ^2^ = 57.84, df = 7, *p* < 0.0001). Both the crosshair and ROC ellipse plots confirmed the heterogeneity present (Fig. S2A-B). Univariate Forest plots of the DOR, posLR, and negLR for each individual analysis are shown in Fig. 5C–E. Bivariate and HSROC models’ summary statistics are provided in Table 5.

**Table 5:** Meta-analysis of diagnostic accuracy for patients with Parkinson’s disease vs multiple system atrophy summary statistics for the bivariate and hierarchal summary receiver operating characteristic (HSROC) models

Model	Variable	Coefficient estimate ± SE (95% CI)
Summary statistic	Sensitivity	0.845 ± 0.042 (0.743–0.912)
	Specificity	0.845 ± 0.027 (0.784–0.891)
	DOR	29.71 ± 10.825 (14.54–60.68)
	posLR	5.44 ± 0.959 (3.85–7.68)
	negLR	0.183 ± 0.050 (0.107–0.312)
	1/negLR	5.46 ± 1.49 (3.20–9.32)
Bivariate	Logit-transformed sensitivity	1.70 ± 0.325 (1.06–2.33)
	Logit-transformed sensitivity variance	1.69 ± 0.207 (1.29–2.10)
	Logit-transformed specificity	0.855 ± 0.448 (0.306–2.39)
	Logit-transformed specificity variance	0.303 ± 0.171 (0.100–0.915)
	Correlation between sensitivity and specificity	-0.140 ± 0.394 (-0.729–0.569)
	AUC (partial AUC)	0.903 (0.866)
HSROC	Lambda (Λ)	3.51 ± 0.385 (2.75–4.26)
	Theta (Θ)	-0.443 ± 0.381 (-1.19–0.304)
	Beta (β)	-0.520 ± 0.382 (-1.36–0.230)
	Variance Λ	0.875 ± 0.488 (0.293–2.61)
	Variance Θ	0.290 ± 0.161 (0.098–0.859)

Heterogeneity (*I*^2^) values exhibited variations based on the approach employed, similar to the aforementioned findings. The Zhou and Dendukuri approach estimated the heterogeneity at 49.7%. The Holling sample size unadjusted approaches reported higher levels of heterogeneity ranging from 90.9% to 92.2%, while adjusted approaches indicated lower levels of heterogeneity ranging from 8.3% to 12%. These findings suggested substantial heterogeneity across the studies, indicating that the variability in the results may not be due solely to random chance but rather to differences among the studies.

The HSROC curve for this model is provided in Fig. 5F. The summary line was found to be distant from the upper left corner, suggesting that measurement of biomarkers in speculative CNS-enriched EVs for distinguishing patients with PD from MSA may not be promising. Moreover, while some studies achieved good sensitivity and specificity, the combined mean for sensitivity and specificity (shown as the circle) indicated that this test only achieved a fair distinguishing ability.

Publication bias assessment using Begg’s correlation (Fig. S3A) and Egger’s regression test (Fig. S3B) revealed no publication bias. However, Deek’s regression indicated that there may be some publication bias (slope = −55.86, SE = 11.35, *t* = −4.92, *p* = 0.0017, Fig. S3C). Further examination using Deek’s funnel plot (Fig. S3D), bagplots (Fig. S3E), and the trim-and-fill method (Fig. S3F) suggested no publication bias.

### PD vs PSP and CBS

As only two studies attempted to differentiate patients with PD from PSP and CBS [16, 24] and one from FTD, PSP, and CBS [17], we used a univariate approach for this analysis.

**Fig. 6:**
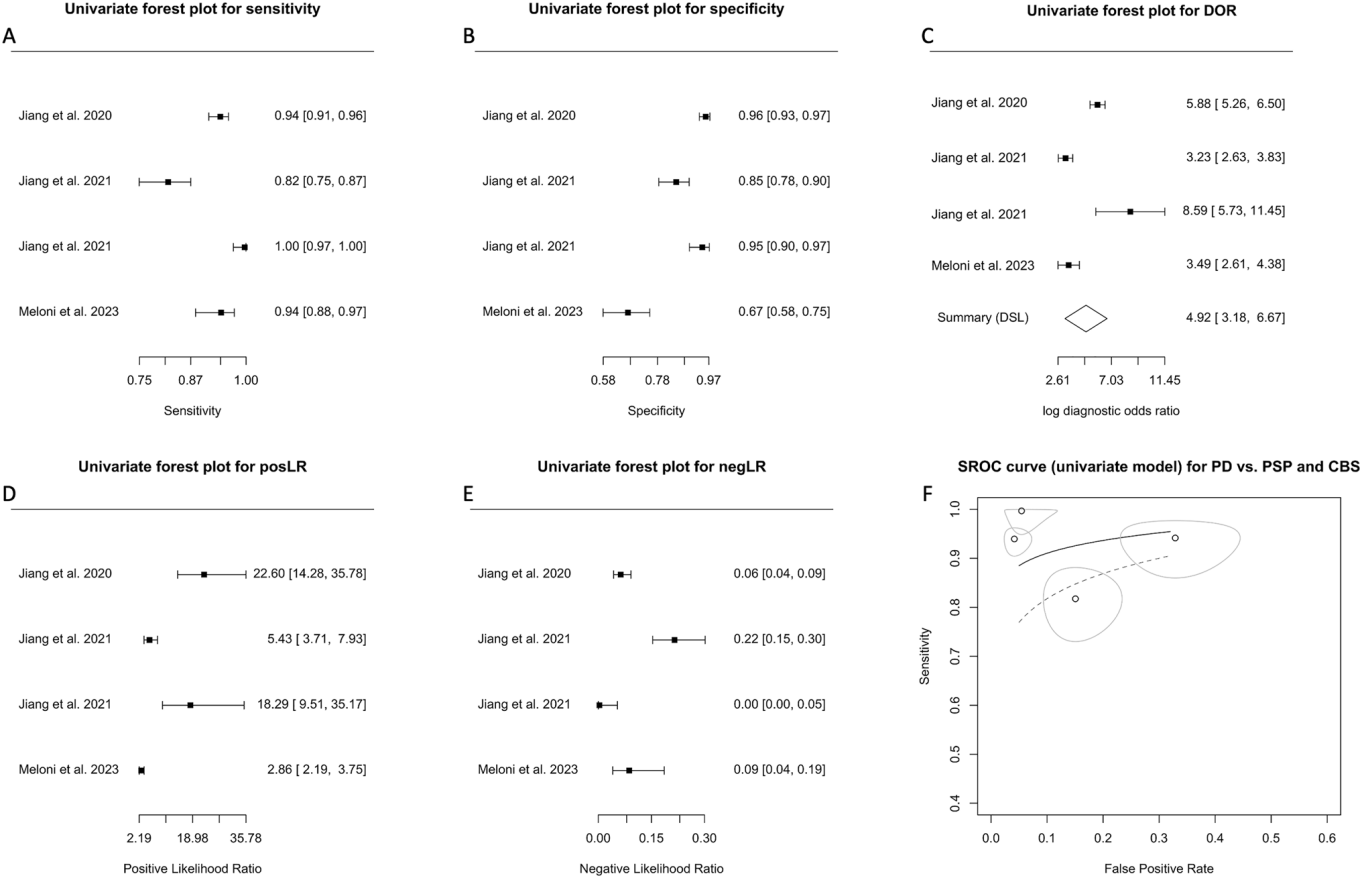
Diagnostic accuracy of biomarkers in speculative CNS-enriched EVs for the differential diagnosis of Parkinson’s disease (PD) from progressive supranuclear palsy (PSP) and corticobasal syndrome (CBS). **A**–**E** Univariate Forest plots for sensitivity, specificity, diagnostic odds ratio (DOR), positive (posLR) and negative (negLR) likelihood ratios, respectively. **F** Summary receiver operating characteristics (SROC) using a univariate model. *CNS* central nervous system; *EVs* extracellular vesicles

Crosshair (Fig. S4A) and ROC ellipse plots (Fig. S4B) suggested low heterogeneity. Forest plots of sensitivity, specificity, DOR, posLR, and negLR are shown in Fig. 6A–E. The model provided an AUC of 0.961 (95% CI: 0.920–1.0), indicating high discriminatory ability. The correlation estimate between sensitivity and FPR was -0.185 (95% CI: -0.973–0.944). The wide confidence interval and the presence of both positive and negative values indicated low precision, high variability, and uncertainty in the correlation estimate. The coefficient θ of 0.041 (95% CI: −0.0058–0.087; plotted as SROC in Fig. 6F) provided support for the utility of this model. The smaller the coefficient θ, the larger the area under the ROC curve, resulting in larger accuracy of the model.

With low heterogeneity (chi-square quality test under heterogeneity: χ^2^ = 4.11, df = 2, *p* value = 0.13), high accuracy and larger overall standardized mean difference of biomarkers in patients with PD vs PSP and CBS [41], measuring biomarkers in speculative CNS-enriched EVs to differentiate patients with PD from PSP and CBS may be promising. However, as the results came only from three studies, two of which are from the same research group [16, 17], interpretation and generalizability are limited. A significant challenge in the field arises from the lack of independent validation across studies, and to combat such issue, it is essential to obtain similar results across different laboratories and cohorts.

Assessment of publication bias using Begg’s correlation (Fig. S5A), Egger’s regression (Fig. S5B), Deek’s regression (Fig. S5C) tests, Deek’s funnel plot (Fig. S5D), bagplots (Fig. S5E), and funnel plots using the trim-and-fill method (Fig. S5F) suggested no publication bias.

### MSA vs control

**Fig. 7:**
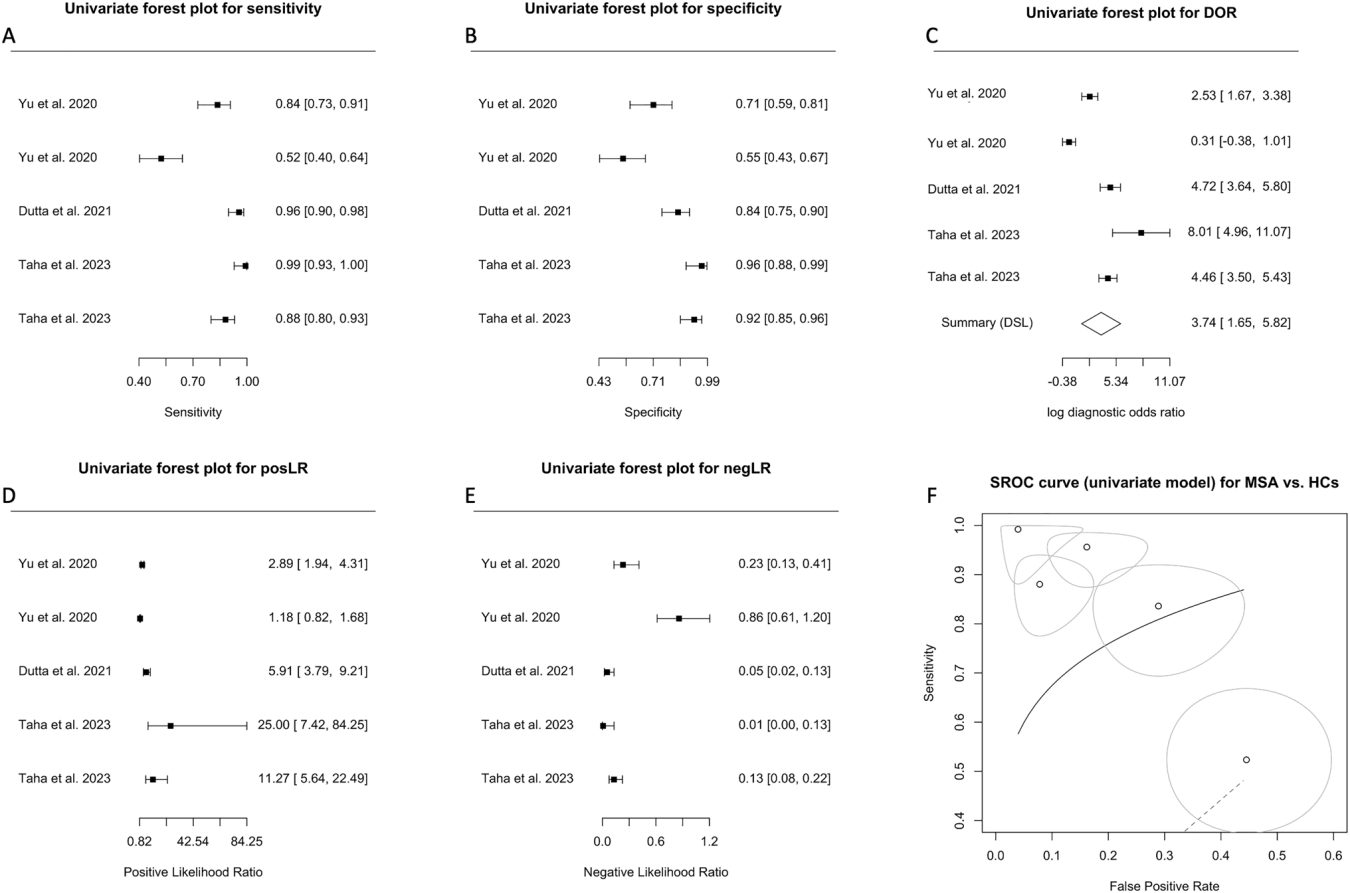
Diagnostic accuracy of biomarkers in speculative CNS-enriched EVs for the differential diagnosis of multiple system atrophy (MSA) from healthy controls (HCs). **A**–**E** Univariate Forest plots for sensitivity, specificity, diagnostic odds ratio (DOR), positive (posLR) and negative (negLR) likelihood ratios, respectively. **F** Summary receiver operating characteristics (SROC) using a univariate model. *CNS* central nervous system; *EVs* extracellular vesicles

Three studies attempted to differentiate patients with MSA from HCs [11, 43, 49] and were analyzed using a univariate approach. The Forest plots for sensitivity, specificity, DOR, posLR, and negLR are shown in Fig. 7A–E. The coefficient θ of 0.17 (95% CI: −0.55–0.89; plotted as SROC in Fig. 7F) indicated that this model is not promising for diagnosing patients with MSA from HCs despite what is reported in the literature [11, 43]. The large coefficient θ suggested smaller AUC and lesser accuracy of this model. Close inspection of the SROC (Fig. 7F) also suggested large variability and heterogeneity, in support of crosshair (Fig. S6A) and ROC ellipse (Fig. S6B) plots.

Assessment of publication bias using Begg’s correlation (Fig. S7A), Egger’s regression (Fig. S7B), Deek’s regression (Fig. S7C) tests, Deek’s funnel plot (Fig. S7D), bagplots (Fig. S7E), and funnel plots using the trim-and-fill method (Fig. S7F) revealed that only Egger’s regression test suspected publication bias.

### Synucleinopathy vs prodromal synucleinopathy

RBD and PAF are recognized as prodromal disorders that are likely to progress and develop into one of the three synucleinopathies [8, 29]. None of the studies with a RBD cohort in the present meta-analysis [17, 35, 48] provided ROC discriminatory models for the disease against PD or DLB except for MSA [17]. Moreover, no study included a PAF cohort, precluding our ability to conduct a meta-analysis.

### RBD vs control

**Fig. 8:**
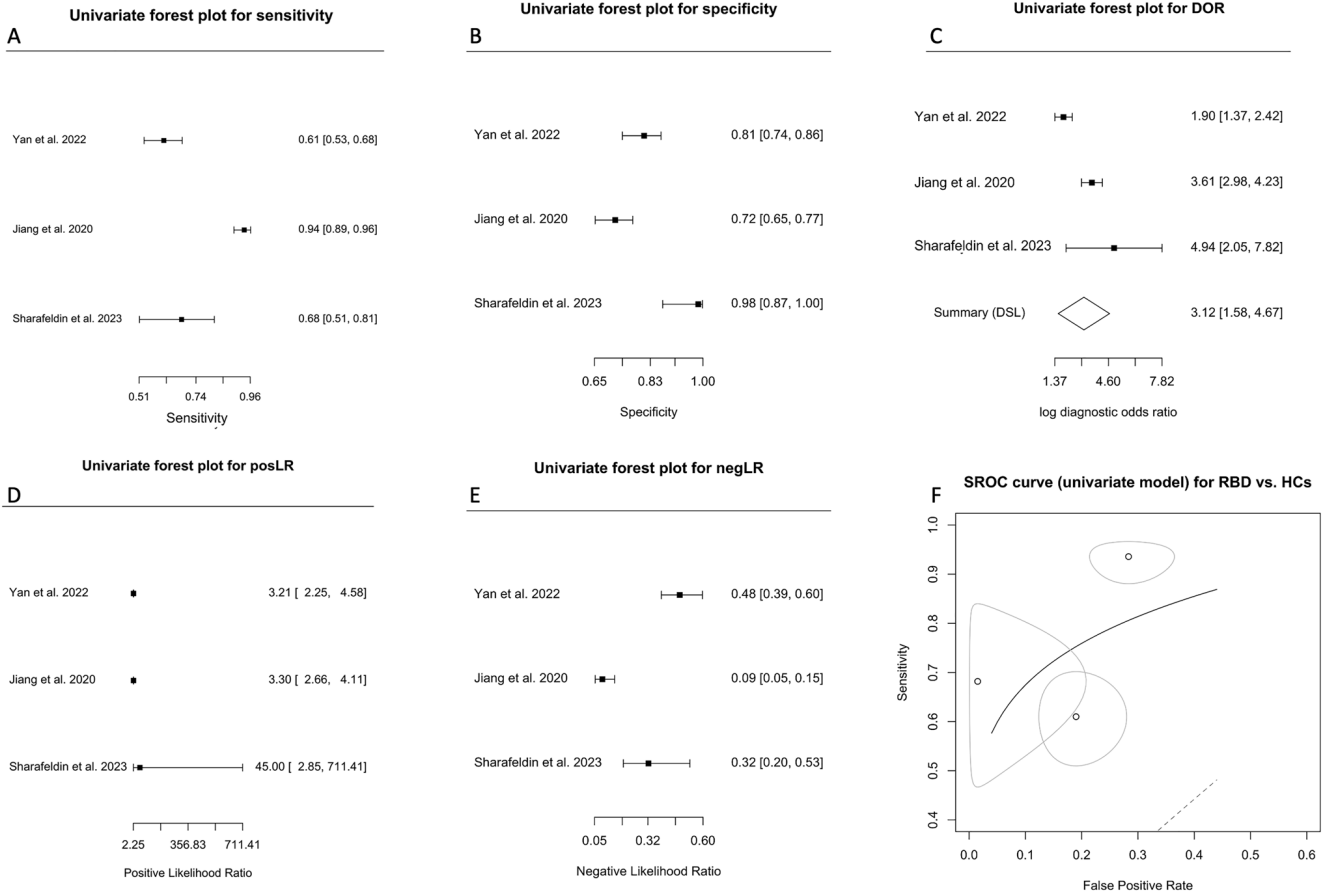
Diagnostic accuracy of biomarkers in speculative CNS-enriched EVs for the differential diagnosis of REM behavior disorder (RBD) from healthy controls (HCs). **A**–**E** Univariate Forest plots for sensitivity, specificity, diagnostic odds ratio (DOR), positive (posLR) and negative (negLR) likelihood ratios, respectively. **F** Summary receiver operating characteristics (SROC) using a univariate model

Three studies evaluated biomarkers in speculative nEVs in an attempt to differentiate patients with RBD vs HCs [17, 35, 48] and were analyzed using a univariate approach. The Forest plots for sensitivity, specificity, DOR, posLR, and negLR are shown in Fig. 8A–E. The large coefficient θ of 0.14 (95% CI: −0.17–0.45; plotted as SROC in Fig. 8F) indicated that this model may not be promising in distinguishing patients with RBD from HCs as it suggested smaller AUC and lesser accuracy. Close inspection of the SROC (Fig. 8F) also suggested large variability and heterogeneity, in support of crosshair (Fig. S8A) and ROC ellipse (Fig. S8B) plots. Since the number of studies was small, with one study not reporting any false positives [35], we did not assess publication bias.

## Discussion

**Fig. 9:**
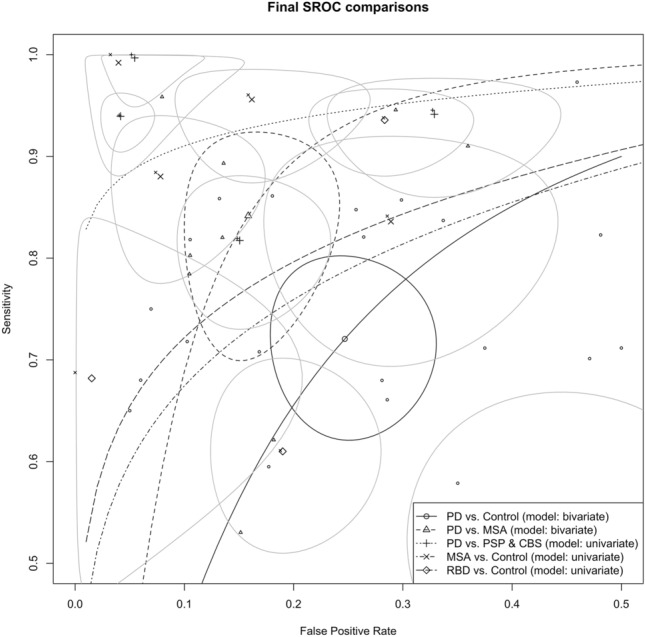
Summary receiver operating characteristic (SROC) comparisons for patients with Parkinson’s disease (PD), multiple system atrophy (MSA), progressive supranuclear palsy (PSP), corticobasal syndrome (CBS) and REM behavior disorder (RBD)

The lack of precise and accurate biomarkers for parkinsonian disorders, including PD, MSA, DLB, PSP, and CBS, often leads to misdiagnoses, hampering patients' ability to receive appropriate and timely care. The inability to predict prodromal disease conversion from RBD and/or PAF to a synucleinopathy further compounds this problem. These challenges are not only distressing for the patients who are left uncertain about their health status and future, but also for the physicians who strive to provide optimal care. Measurement of biomarkers in speculative CNS-enriched EVs isolated from the blood has been popular due to their hypothesized ability to contain cell-state-specific biomarkers and traverse the blood-brain barrier to the peripheral circulation. The current meta-analysis encompassed 18 studies [1, 5, 11, 16–18, 24, 25, 35, 37–39, 43, 46, 48–50, 52] with 1695 patients with PD, 253 with MSA, 21 with DLB, 172 with PSP, 152 with CBS, 189 with RBD and 1288 HCs (Table 1) and aimed to evaluate the diagnostic accuracy of biomarkers in speculative CNS-enriched EVs for parkinsonian disorders (Fig. 9).

Studies (*n* = 16) attempting to differentiate patients with PD from HCs exhibited considerable variability in sensitivity (Fig. 2A) and specificities, (Fig. 2B), indicating potential methodological inconsistencies among them. The analysis showed that while biomarkers in speculative CNS-enriched EVs achieved a fair ability in distinguishing patients with PD from HCs (Fig. 2F, Table 3), the results were plagued by high heterogeneity and potential publication bias (Fig. 3A–F), casting doubts on the reliability of these findings. Furthermore, our examination using the trim-and-fill method suggested that smaller studies with lower or non-significant diagnostic odds ratios (*n* = 5) have been less likely to be published (white circles in Fig. 3F). This revealed a substantial overestimation of the diagnostic utility of biomarkers in speculative CNS-enriched EVs for patients with PD.

Comparing the diagnostic accuracy of biomarkers in speculative CNS-enriched EVs isolated from the plasma (Table S4) vs serum (Table S5), suggested that serum may be superior in accuracy (Fig. 4A). However, three and two studies out of 7 using serum were from the same research group while all studies using plasma were mostly from unique research groups, suggesting possible bias in studies using serum. Further comparisons by the anti-L1CAM antibody clone UJ127 (Table S6) vs 5G3 (Table S7) did not reveal substantial differences with large overlap in the confidence intervals, though studies using the 5G3 clone obtained a slightly higher accuracy (Fig. 4B). Comparison of studies based on quantification methodology (Fig. 4C, Table S8) revealed that ELISA achieved the highest diagnostic accuracy followed by ECLIA and bead-based arrays (i.e., Simoa, Luminex). We also noted that general EVs [42] obtained better diagnostic accuracy and less publication than speculative CNS-enriched EVs (Table 4) as the trim-and-fill method estimated 2 missing studies out of 21 vs 5 out of 16 for the former and latter, respectively.

On the other hand, six studies [11, 16, 17, 43, 46, 49] attempted to differentiate patients with PD from MSA and provided mixed results. The analysis (Table 5) revealed wide-ranging values for sensitivity (Fig. 5A), specificity (Fig. 5B), and DOR (Fig. 5C), underlining the significant variability among these studies. Although the collective AUC was 0.903 (Fig. 5F), suggesting a reasonable diagnostic test's discriminatory capacity, the substantial heterogeneity in the results raises concerns about the reliability of the findings.

Only three studies [16, 17, 24] attempted to distinguish patients with PD from those with PSP and CBS. The results, while promising with an AUC = 0.961 (Fig. 6F), are undermined by wide confidence intervals and both positive and negative values in the correlation estimate between sensitivity and FPR (−0.185, 95% CI: −0.973–0.944). This variability indicated uncertainty in the reliability of these findings. The studies exhibited low heterogeneity, which usually strengthens the findings; however, considering two of the three studies originated from the same research group [16, 17], this limited pool restricted the conclusions' generalizability. More diverse research is required to confirm these results and establish the potential of biomarkers in speculative CNS-enriched EVs in differentiating patients with PD from PSP and CBS.

Three studies attempted to differentiate patients with MSA from HCs, but despite prior reports of successful differentiation [11, 43], our analysis suggested that this approach may not be as promising. A high coefficient θ (0.17, 95% CI: −0.55–0.89, Fig. 7F), indicating smaller AUC and lesser accuracy, along with large variability and heterogeneity raises concerns about the reliability of this diagnostic approach.

The prodromal disorders RBD and PAF are considered to eventually convert into one of the three synucleinopathies: PD, MSA, and/or DLB. However, none of the studies that included an RBD cohort [17, 35, 48] provided a ROC discriminatory model for the disease against patients with PD or DLB, except for MSA [36], while no study to date examined biomarkers in speculative CNS-enriched EVs for the prodromal disorder PAF. The attempt to differentiate patients with RBD from HCs in three studies [17, 35, 48] also appears unpromising, as suggested by the large coefficient θ (0.14, 95% CI: -0.17–0.45; Fig. 8F) indicating smaller AUC and lesser accuracy, along with significant variability and heterogeneity.

Notably, one critical challenge is that studies measuring biomarkers in speculative CNS-enriched EVs suffer from a failure of independent validation and replication, even when the same methodology is employed. There is also a lack of standardization of pre-analytical factors in obtaining speculative CNS-enriched EVs despite them being highly sensitive to these pre-analytical factors [40], which further complicates the generalizability of such a test in the clinic.

Importantly, most studies did not adequately detail information concerning pharmacological treatments, such as type, duration and dosage, which are likely to alter the EVs signature. There was also a notable absence of data on race/ethnicity and potential comorbidities, all of which can influence the outcomes. It is imperative that studies using speculative CNS-enriched EVs or general EVs provide a thorough and detailed methodology of blood handling through the EV-TRACK platform [7] as previously reported [40–42] along with comprehensive information on the pre-analytical factors. These include but are not limited to fasting status before blood collection, the time of day when blood was collected, the duration of the blood collection process, the needle size used, the specific method and duration for blood layer separation, and the type of tube utilized. Additionally, considerations such as the nature of transport, whether the tube was oriented vertically or horizontally, the chosen anticoagulation agent mixed with plasma, centrifugation techniques, the number of freeze–thaw cycles, platelet-depletion processes, storage conditions (including time and temperature), defibrinization treatments, and the methodologies for freezing EVs or EV lysates after isolation and lysis should also be meticulously documented [40].

In the broader landscape of clinical practice, this meta-analysis uncovers crucial concerns. Though individual studies may seem promising, the current meta-analysis suggested otherwise. Diverse methodologies and variations among the studies using speculative CNS-enriched EVs challenge the reliability of these findings for everyday clinical application. Most critically, such inconsistencies hampers the successful development of a dependable biomarker for parkinsonian disorders. Finding such biomarkers could serve multifaceted roles: diagnosing the diseases, providing prognosis insights, distinguishing the mamong one another or from HCs, tracking disease progression, monitoring and anticipating how a patient might respond to treatment, initial screening, evaluating patient risk, stratifying patients in clinical trials, interpreting drug behavior and responses in the body, discovering the origins and mechanisms of the disorder, identifying environmental triggers or exposures, and playing a key role as primary or alternative measures in clinical research trials. Moreover, having a reliable biomarker would alleviate the undue stress and concerns faced by patients and their families due to uncertainties in diagnosis or prognosis.

As the search for reliable biomarkers in parkinsonian disorders persists, it becomes evident that a more standardized and rigorous approach is imperative in the field. As we move forward, greater emphasis should be placed on improving study design and minimizing bias, enhancing the comparability and reproducibility of findings, and addressing the heterogeneity in the results. Current efforts by the International Society for Extracellular Vesicles (ISEV) [44] and others [7, 15] aim toward more rigorous reporting and standardization to enhance accuracy and reproducibility of research utilizing EVs.

## Conclusion

Our comprehensive meta-analysis underscores current limitations and challenges associated with the use of speculative CNS-enriched EVs as diagnostic biomarkers for parkinsonian disorders. The significant methodological inconsistencies across studies, combined with high levels of heterogeneity and potential publication bias, considerably undermine the reliability of these findings. Furthermore, the occasional signs of diagnostic promise are frequently offset by the presence of considerable variability, publication bias, and the lack of independent validation across different research groups. The absence of standardized protocols for pre-analytical factors, which are critical in determining the accuracy of EV-based biomarkers, further compounds these issues. All these aspects culminate in a rather sobering picture, suggesting that this approach may not provide the anticipated breakthrough in the diagnosis of parkinsonian disorders. As we navigate through the complexities of these debilitating diseases, it is becoming increasingly clear that we may need to re-evaluate our strategies, either by adopting more rigorous standardization and reporting [15] as suggested through current efforts by ISEV [44] and others [7] or exploring alternative avenues for effective biomarker discovery. While the journey ahead may be challenging, our continued pursuit of this endeavor remains crucial in transforming the landscape of discovering biomarkers for parkinsonian disorders diagnosis and management.

### Supplementary Information

Below is the link to the electronic supplementary material.

Supplementary file1 (PDF 1224 KB)

## Data Availability

Not applicable.

## Abbreviations

AUC: Area under the curve
aEVs: Astrocyte extracellular vesicles
CBS: Corticobasal syndrome
CI: Confidence interval
CNS: Central nervous system
DLB: Dementia with Lewy bodies
DOR: Diagnostic odds ratio
ECLIA: Electrochemilumiscence ELISA
EDTA: Ethylenediaminetetraacetic acid
ELISA: Enzyme-linked immunosorbent assay
EVs: Extracellular vesicles
FPR: False-positive rate
FTD: Frontotemporal dementia
HCs: Healthy controls
HSROC: Hierarchical summary receiver operating characteristic
*I*^2^: Heterogeneity statistic
L1CAM: L1 cell adhesion molecule
MSA: Multiple system atrophy
nEVs: Neuronal extracellular vesicles
negLR: Negative likelihood ratio
oEVs: Oligodendroglial extracellular vesicles
PAF: Pure autonomic failure
PD: Parkinson’s disease
posLR: Positive likelihood ratio
PSP: Progressive supranuclear palsy
PRISMA: Preferred reporting items for systematic reviews and meta-analyses
QUADAS-2: Quality assessment for diagnostic accuracy studies
RBD: REM behavior disorder
ROC: Receiver operating characteristic
Simoa: Single molecule array
SROC: Summary receiver operating characteristics
χ^2^: Chi-square
α-Syn: α-Synuclein
WB: Western blot
