# Supplementary material for: Analysis of biomarkers in speculative CNS-enriched extracellular vesicles for parkinsonian disorders: a comprehensive systematic review and diagnostic meta-analysis
Source: J Neurol. 2023 Dec 16;271(4):1680–706. doi: 10.1007/s00415-023-12093-3 (PMC10973014; doi:10.1007/s00415-023-12093-3)
Supplement: Supplementary file 1 — Supplementary file1 (PDF 1224 KB) [file 415_2023_12093_MOESM1_ESM.pdf]

Table S1. Complete Search Strategy using PUBMED and EMBASE

PUBMED	(((Parkinson's disease) AND (extracellular vesicle OR exosome)) AND (Diagnosis)
EMBASE	(parkinsons:ti,ab,kw AND disease:ti,ab,kw OR (multiple:ti,ab,kw AND system:ti,ab,kw AND atrophy:ti,ab,kw) OR (lewy:ti,ab,kw AND body:ti,ab,kw AND dementia:ti,ab,kw) OR (corticobasal:ti,ab,kw AND syndrome:ti,ab,kw) OR (progressive:ti,ab,kw AND supranuclear:ti,ab,kw AND palsy:ti,ab,kw)) AND (neuronal:ti,ab,kw AND extracellular:ti,ab,kw AND vesicles:ti,ab,kw OR evs:ti,ab,kw OR exosomes:ti,ab,kw OR (oligodendrocyte:ti,ab,kw AND extracellular:ti,ab,kw AND vesicles:ti,ab,kw) OR (oligodendrocyte:ti,ab,kw AND evs:ti,ab,kw) OR (astrocyte:ti,ab,kw AND extracellular:ti,ab,kw AND vesicles:ti,ab,kw) OR (astrocyte:ti,ab,kw AND evs:ti,ab,kw) OR (microglia:ti,ab,kw AND extracellular:ti,ab,kw AND vesicles:ti,ab,kw) OR (microglia:ti,ab,kw AND evs:ti,ab,kw)) AND (cns:ti,ab,kw OR brain:ti,ab,kw OR (central:ti,ab,kw AND nervous:ti,ab,kw AND system:ti,ab,kw) OR ('cns originating':ti,ab,kw AND evs:ti,ab,kw) OR ('cns derived':ti,ab,kw AND evs:ti,ab,kw))

Table S2. Rubric for QUADAS-2.

DOMAIN	DOMAIN 1<br>Patient selection	DOMAIN 2<br>Index test(s)	DOMAIN 3<br>Reference standard	DOMAIN 4<br>Flow and timing
Description	Describe methods of patient selection.<br>Describe included patients (prior testing, presentation, intended use of index test and setting).	Describe the index test and how it was conducted and interpreted.	Describe the reference standard and how it was conducted and interpreted.	Describe any patients who did not receive the index test(s) and/or reference standard or who were excluded from the 2x2 table (refer to flow diagram).<br>Describe the time interval and any interventions between index test(s) and reference standard.
Signaling questions (*yes/no/unclear*)	Was a case-control design avoided?<br>Was a consecutive or random sample of patients enrolled?<br>Did the study avoid inappropriate exclusions?	Were the index test results interpreted without knowledge of the results of the reference standard?<br>If a threshold was used, was it pre-specified?	Is the reference standard likely to correctly classify the target condition?<br>Were the reference standard results interpreted without knowledge of the results of the index test?	Was there an appropriate interval between index test(s) and reference standard?<br>Did all patients receive a reference standard?<br>Did patients receive the same reference standard?<br>Were all patients included in the analysis?
Overall judgement	Included studies only were considered eligible if they determined the levels of biomarkers in CNS-originating extracellular vesicles (either neuronal (nEVs) or oligodendroglial (oEVs) in at least Parkinson's disease (low risk of bias) and one other parkinsonian disorder or healthy controls	Even though knowledge of the diagnosis may affect the interpretation of the diagnostic test results, measuring α-syn in nEVs or oEVs is an objective method that should not be influenced by the diagnosis.<br>This is considered a low risk of bias,	To diagnose PD, the standard used was the United Kingdom Parkinson's Disease Society Brain Bank or the MDS clinical diagnostic criteria were used. To diagnose MSA, the second consensus statement on the	All patients were classified according to the appropriate diagnostic criteria (see Domain 3).<br>Low risk of bias was considered if all the questions were answered "yes".<br>Unclear risk of bias was considered if they did not cover the time interval between clinical diagnosis and index test.

	Unclear risk of bias was considered in the absence of information on consecutive patient enrollment.<br><br>High risk of bias was based on the absence of information on consecutive patient enrollment and any unexplained or suspected exclusions.	even if blinding was not used.	diagnosis of multiple system atrophy was used To diagnose DLB, the fourth consensus report of the DLB consortium was used. To diagnose PSP, the NINDS-SPSP International workshop or the movement disorder society criteria were used To diagnose CBS, the criteria for the diagnosis of corticobasal degeneration were used. The clinical diagnoses were established before the index test (low risk of bias).<br><br>If the diagnosis was based on symptoms/signs without consultation of the diagnostic criteria listed above, the study was rated as "unclear risk of bias".	High risk of bias was considered if the study excluded any of the participants from the analysis.

Concerns regarding applicability (*High/low/unclear*)	Are there concerns that the included patients do not match the review question?	Are there concerns that the index test, its conduct, or interpretation differs from the review question?	Are there concerns that the target condition as defined by the reference standard does not match the review question?	Could the patient flow have introduced bias?
Applicability: Overall judgement	As reported in the inclusion and eligibility criteria, the clinical diagnoses for the diseases were based on established diagnostic criteria (see Domain 3). Therefore, all studies were rated as “low concern/high applicability”	If the protein concentrations were determined using a standard calibration curve, the risk of bias was considered low. If this information was not provided, the risk of bias was deemed unclear.<br><br>The usage of in-house developed tests was considered “high concern/low applicability”	Because all the studies used internationally recognized criteria for their assessments, the risk of bias was considered low.	NA

Table S3. Risk of Bias assessment according to the QUADAS-2 per study included in the meta-analyses.

	Bias				Applicability concern		
	Patient selection	Index text	Reference standard	Flow and timing	Patient selection	Index text	Reference standard
**CNS-Originating EVs**							
Shi et al. 2014	U	L	L	L	L	H	L
Shi et al. 2016	U	L	L	L	L	L	L
Zhao et al. 2019	U	L	L	L	L	L	L
Si et al. 2019	U	L	L	L	L	L	L
Jiang et al. 2020	U	L	L	L	L	L	L
Niu et al. 2020	U	L	L	L	L	L	L
Zou et al. 2020	U	L	L	L	L	L	L
Yu et al. 2020	U	L	L	L	L	H	L
Agliardi et al. 2021	U	L	L	L	L	L	L
Jiang et al. 2021	U	L	L	L	L	L	L
Duta et al. 2021	U	L	L	L	L	L	L
Yan et al. 2022	U	L	L	L	L	H	L
Meloni et al. 2023	U	L	L	L	L	L	L
Taha et al. 2023	U	L	L	L	L	H	L
Sharafeldin et al. 2023	U	L	L	L	L	H	L
Jiao et al. 2023	U	L	L	L	L	L	L
Wang et al. 2023		L	L	L	L	L	L
Chen et al. 2023		L	L	L	L	L	L

Table S4. Meta-analysis of diagnostic accuracy for patients with Parkinson's disease vs. healthy control summary statistics for the bivariate and hierarchal summary receiver operating characteristic (HSROC) models using CNS-originating EVs isolated from plasma.

Model	Variable	Coefficient Estimate ± SE (95% CI)
**Summary Statistic**		
	Sensitivity	0.720 ± 0.065 (0.578 – 0.828)
	Specificity	0.705 ± 0.047 (0.605 – 0.788)
	DOR	6.15 ± 2.42 (2.84 – 13.32)
	posLR	2.44 ± 0.448 (1.70 – 3.50)
	negLR	0.397 ± 0.096 (0.247 – 0.637)
	1/negLR	2.52 ± 0.608 (1.57 – 4.04)
**Bivariate**		
	Logit-Transformed Sensitivity	0.897 ± 0.299 (0.312 – 1.483)
	Logit-Transformed Sensitivity Variance	0.851 ± 0.209 (0.441 – 1.261)
	Logit-Transformed Specificity	1.18 ± 0.514 (0.506 – 2.775)
	Logit-Transformed Specificity Variance	0.565 ± 0.240 (0.245 – 1.300)
	Correlation between sensitivity and specificity	0.024 ± 0.286 (-0.491 – 0.527)
	AUC (partial AUC)	0.755 (0.612)
**HSROC**		
	Lambda (Λ)	1.83 ± 0.384 (1.08 – 2.59)
	Theta (Θ)	-0.129 ± 0.233 (-0.586 – 0.329)
	Beta (β)	-0.364 ± 0.314 (-1.16 – 0.250)
	Variance Λ	1.78 ± 0.758 (0.774 – 4.10)
	Variance Θ	0.438 ± 0.191 (0.186 – 1.031)

Table S5. Meta-analysis of diagnostic accuracy for patients with Parkinson's disease vs. healthy control summary statistics for the bivariate and hierarchal summary receiver operating characteristic (HSROC) models using CNS-originating EVs isolated from serum.

Model	Variable	Coefficient Estimate ± SE (95% CI)
**Summary Statistic**		
	Sensitivity	0.746 ± 0.040 (0.660 – 0.816)
	Specificity	0.826 ± 0.031 (0.756 – 0.880)
	DOR	13.94 ± 3.94 (8.01 – 24.27)
	posLR	4.29 ± 0.781 (3.01 – 6.13)
	negLR	0.308 ± 0.048 (0.227 – 0.418)
	1/negLR	3.25 ± 0.508 (2.39 – 4.41)
**Bivariate**		
	Logit-Transformed Sensitivity	1.07 ± 0.210 (0.663 – 1.49)
	Logit-Transformed Sensitivity Variance	1.56 ± 0.219 (1.13 – 1.99)
	Logit-Transformed Specificity	0.369 ± 0.192 (0.133 – 1.02)
	Logit-Transformed Specificity Variance	0.367 ± 0.222 (0.112 – 1.20)
	Correlation between sensitivity and specificity	-0.163 ± 0.373 (-0.724 – 0.527)
	AUC (partial AUC)	0.851 (0.734)
**HSROC**		
	Lambda (Λ)	2.64± 0.291 (2.06 – 3.21)
	Theta (Θ)	-0.244 ± 0.292 (-0.817 – 0.329)
	Beta (β)	-0.002 ± 0.395 (-0.00 – 0.996)
	Variance Λ	1.81 ± 0.753 (0.804 – 4.09)
	Variance Θ	0.391 ± 0.172 (0.166 – 0.925)

Table S6. Meta-analysis of diagnostic accuracy for patients with Parkinson's disease vs. healthy control summary statistics for the bivariate and hierarchal summary receiver operating characteristic (HSROC) models using neuronal extracellular vesicles (EVs) isolated using the anti-L1CAM antibody clone UJ127.

Model	Variable	Coefficient Estimate ± SE (95% CI)
**Summary Statistic**		
	Sensitivity	0.714 ± 0.057 (0.590 – 0.812)
	Specificity	0.724 ± 0.041 (0.638 – 0.797)
	DOR	6.55 ± 2.36 (3.23 – 13.27)
	posLR	2.59 ± 0.451 (1.84 – 3.64)
	negLR	0.395 ± 0.084 (0.261 – 0.599)
	1/negLR	0.714 ± 0.057 (0.590 – 0.812)
**Bivariate**		
	Logit-Transformed Sensitivity	0.913 ± 0.279 (0.366 – 1.461)
	Logit-Transformed Sensitivity Variance	0.966 ± 0.204 (0.565 – 1.366)
	Logit-Transformed Specificity	1.10 ± 0.463 (0.483 – 2.51)
	Logit-Transformed Specificity Variance	0.560 ± 0.246 (0.237 – 1.33)
	Correlation between sensitivity and specificity	0.097 ± 0.278 (-0.425 – 0.570)
	AUC (partial AUC)	0.770 (0.628)
**HSROC**		
	Lambda (Λ)	1.91 ± 0.355 (1.22 – 2.61)
	Theta (Θ)	-0.186 ± 0.209 (-0.596 – 0.224)
	Beta (β)	-0.338 ± 0.303 (-0.931 – 0.256)
	Variance Λ	1.72 ± 0.694 (0.783 – 3.794)
	Variance Θ	0.355 ± 0.152 (0.153 – 0.820)

Table S7. Meta-analysis of diagnostic accuracy for patients with Parkinson's disease vs. healthy control summary statistics for the bivariate and hierarchal summary receiver operating characteristic (HSROC) models using neuronal extracellular vesicles (EVs) isolated using the anti-L1CAM antibody clone 5G3.

Model	Variable	Coefficient Estimate ± SE (95% CI)
**Summary Statistic**		
	Sensitivity	0.731 ± 0.049 (0.625 – 0.816)
	Specificity	0.792 ± 0.046 (0.689 – 0.868)
	DOR	10.37 ± 3.16 (5.71 – 18.85)
	posLR	3.52 ± 0.731 (2.342 – 5.29)
	negLR	0.339 ± 0.058 (0.242 – 0.475)
	1/negLR	2.95 ± 0.505 (2.10 – 4.12)
**Bivariate**		
	Logit-Transformed Sensitivity	1.0 ± 0.249 (0.512 – 1.49)
	Logit-Transformed Sensitivity Variance	1.34 ± 0.278 (0.794 – 1.884)
	Logit-Transformed Specificity	0.424 ± 0.245 (0.137 – 1.31)
	Logit-Transformed Specificity Variance	0.528 ± 0.311 (0.167 – 1.68)
	Correlation between sensitivity and specificity	-0.392 ± 0.358 (-0.847 – 0.392)
	AUC (partial AUC)	0.822 (0.746)
**HSROC**		
	Lambda (Λ)	2.32 ± 0.306 (1.72 – 2.92)
	Theta (Θ)	-0.105 ± 0.301 (-0.695 – 0.485)
	Beta (β)	0.110 ± 0.388 (0.280 – 0.871)
	Variance Λ	0.575 ± 0.370 (0.163 – 2.030)
	Variance Θ	0.330 ± 0.183 (0.111 – 0.980)

Table S8. Meta-analysis of diagnostic accuracy for patients with Parkinson's disease vs. healthy control summary statistics for the bivariate and hierarchal summary receiver operating characteristic (HSROC) based on quantification methodology of biomarkers in CNS-originating extracellular vesicles (EVs). The sensitivity, specificity, pooled area under the curve (AUC) and partial AUC, focusing on a specific range of false positive rates (FPR), are obtained using the bivariate model. The diagnostic odds ratio (DOR) is obtained from the HSROC model. EV — extracellular vesicles. CNS — central nervous system. SE – standard error. Bead-based array refers to either Simoa or Luminex. ECLIA – electrochemilumiscence ELISA. ELISA – Enzyme-linked immunosorbent assay.

**Quantification Method**	**Mean Sensitivity (95% CI)**	**Mean Specificity (95% CI)**	**Pooled AUC (partial AUC))**	**Mean DOR ± SE (95% CI)**
**Bead-based array**	66.0% (60.9 – 70.9%)	64.7% (55.1 – 73.7%)	0.692 (0.638)	3.97 ± 1.30 (2.35 – 6.68)
**ECLIA**	72.7% (54.6 – 85.5%)	79.0% (69.0 – 86.5%)	0.823 (0.701)	11.55 ± 0.47 (4.59 – 28.9)
**ELISA**	76.9% (68.0 – 84.0%)	77.2% (64.9 – 86.1%)	0.829 (0.764)	11.71 ± 0.34 (6.05 – 22.69)

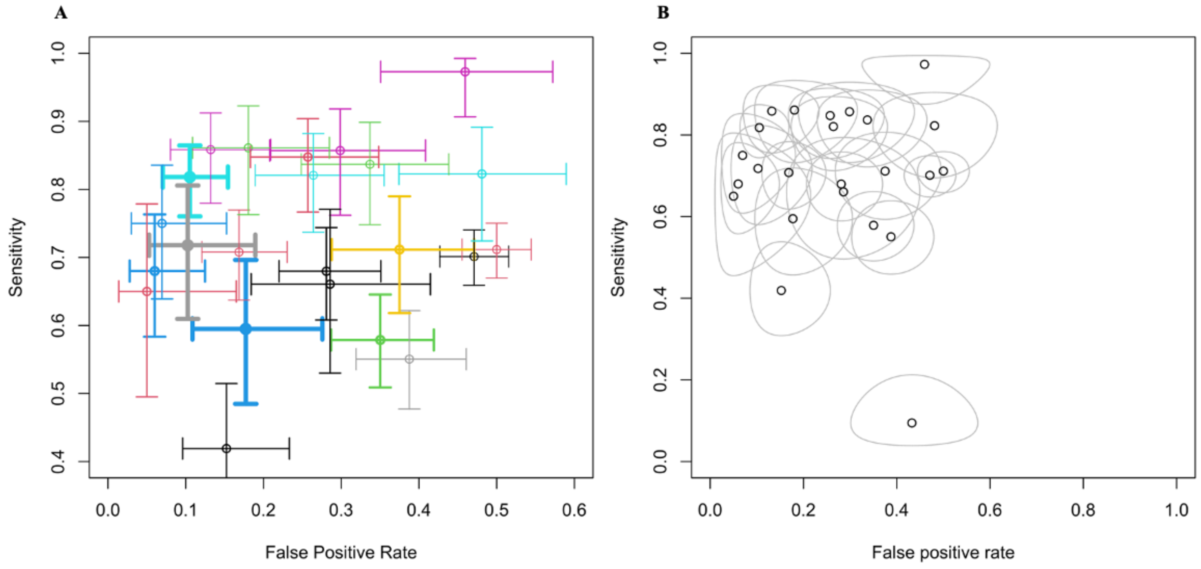

FIGURE S1. (A) Crosshair and **(B)** Receiver operating characteristics plots for studies using biomarkers in putative CNS-originating EVs for the differential diagnosis of patients with Parkinson’s disease from healthy controls.

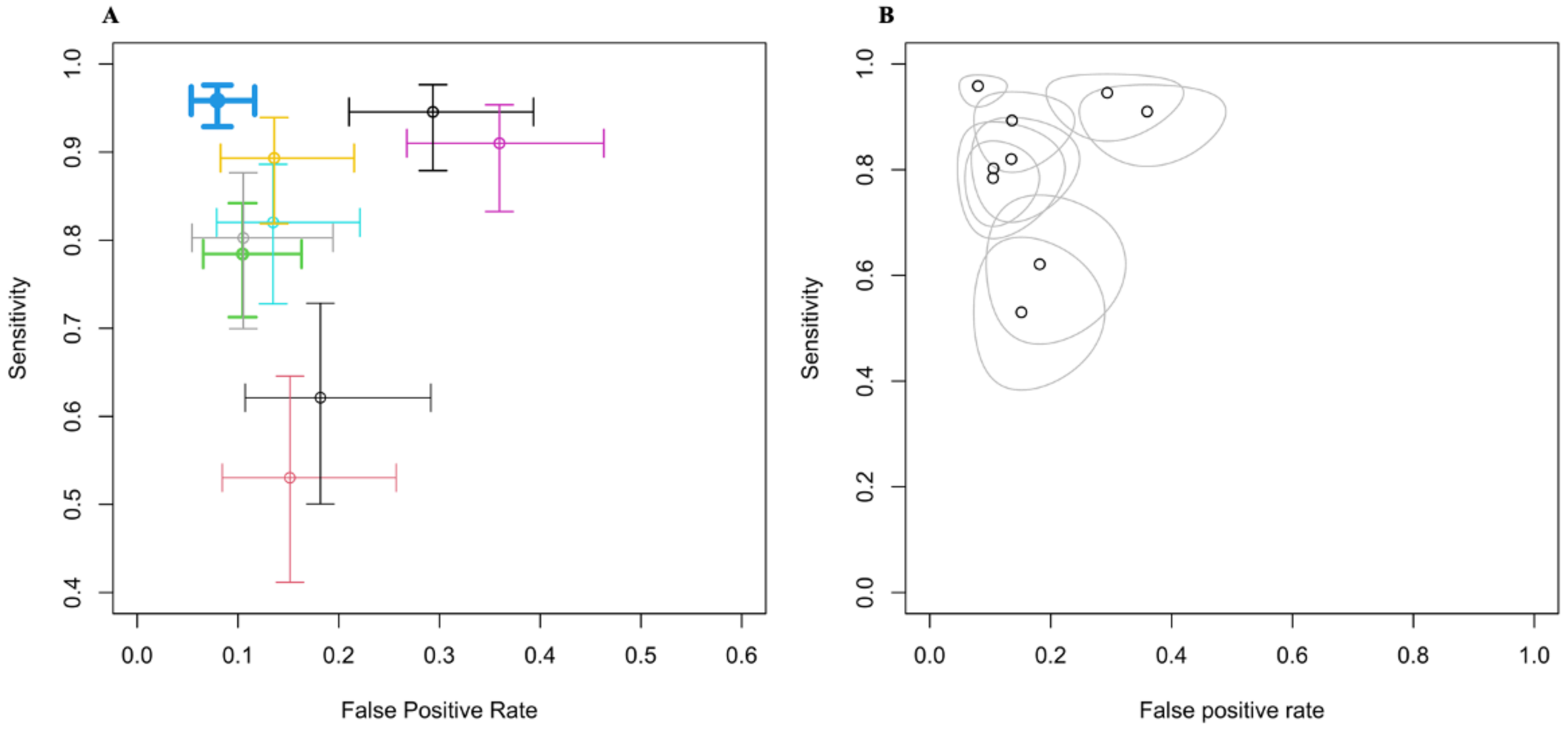

FIGURE S2. (A) Crosshair and **(B)** Receiver operating characteristics ellipse plots for studies using biomarkers in putative CNS-originating EVs for the differential diagnosis of patients with Parkinson's disease from multiple system atrophy.

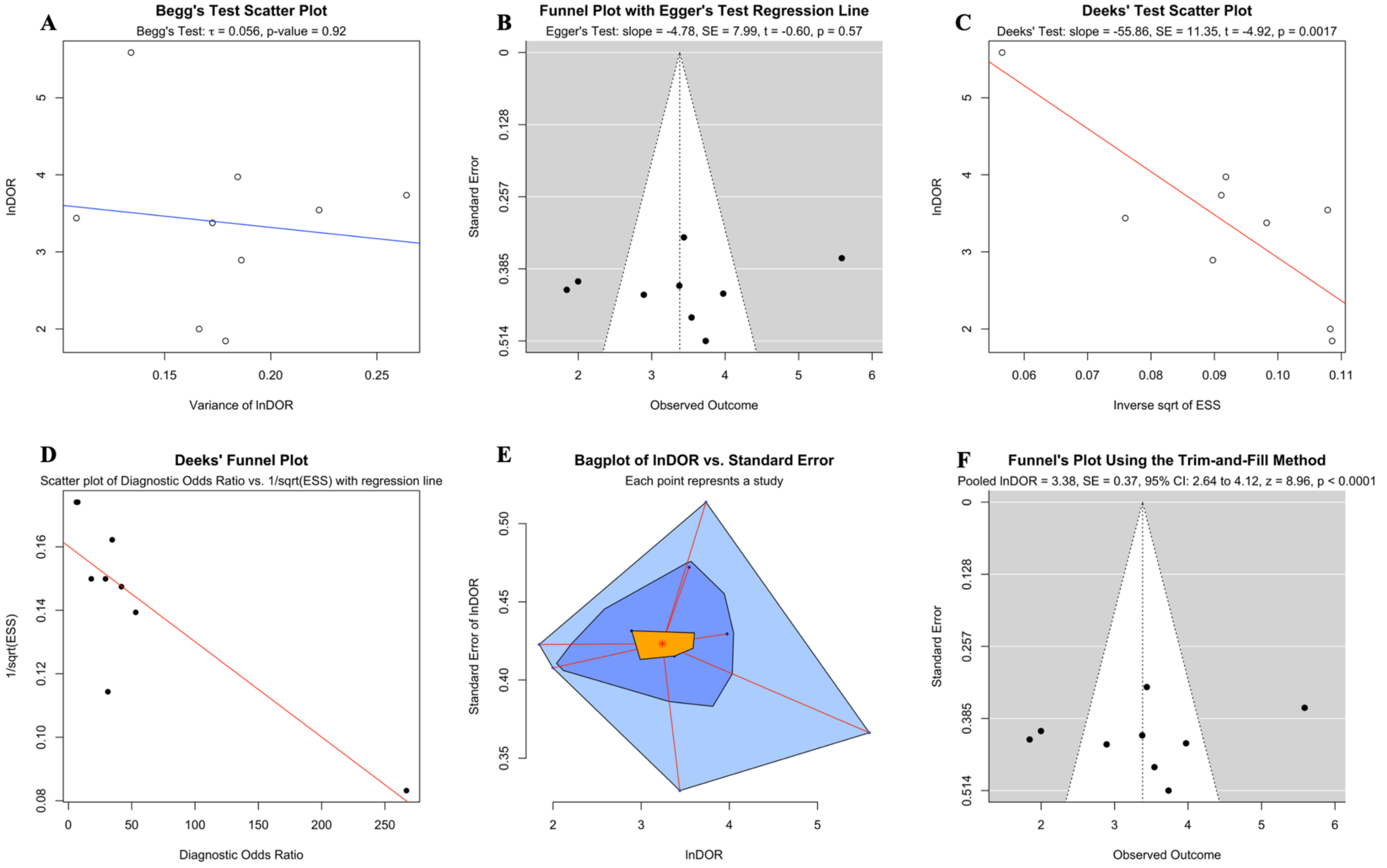

FIGURE S3. Publication bias assessed using **(A)** Begg's correlation, **(B)** Egger's regression, **(C)** Deek's regression, **(D)** Deek's funnel plot, **(E)** A bagplot and **(F)** Funnel plot after application of the trim-and-fill method for biomarkers in putative CNS-originating EVs for the differential diagnosis of Parkinson's disease from multiple system atrophy. Collectively, they suggested no presence of publication bias.

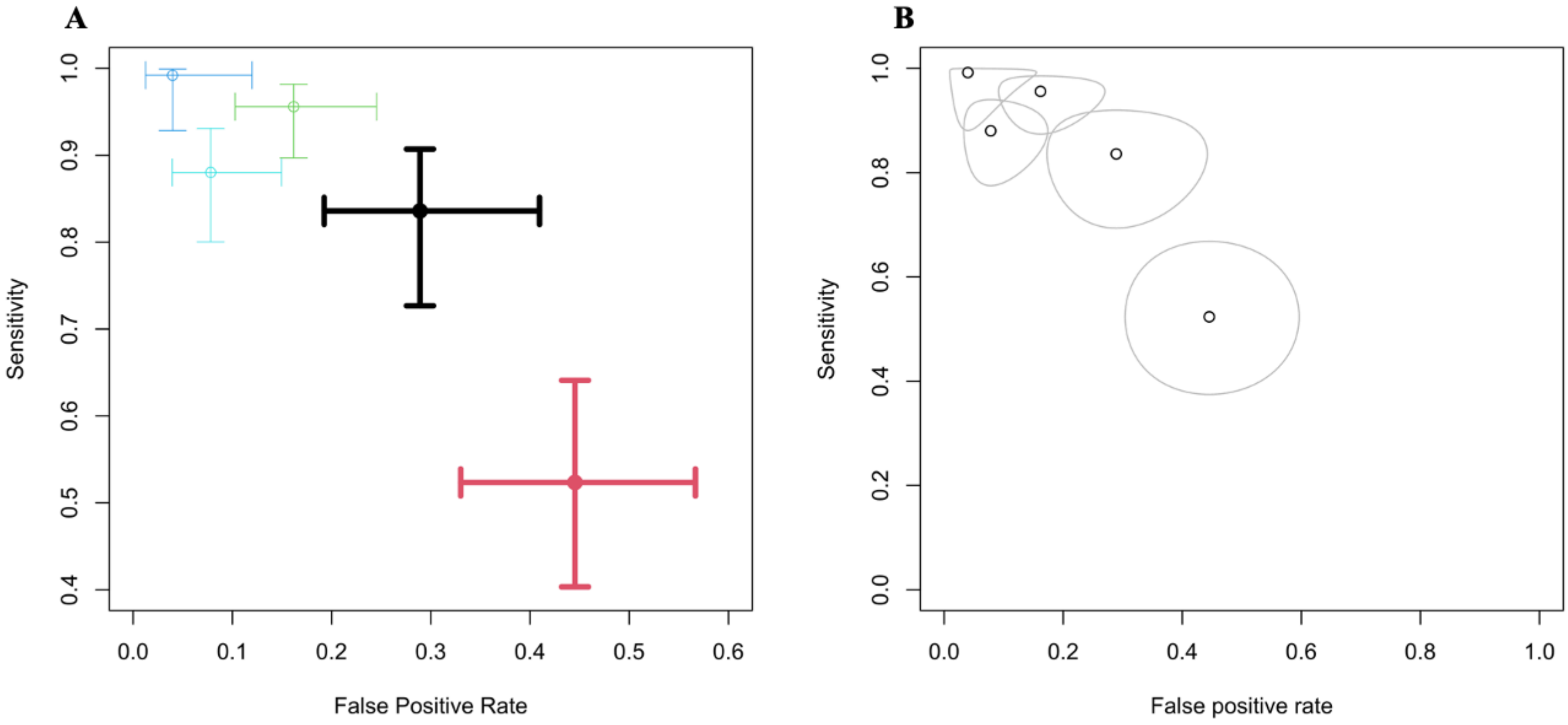

FIGURE S4. (A) Crosshair and **(B)** Receiver operating characteristics ellipse plots for studies using biomarkers in putative CNS-originating EVs for the differential diagnosis of patients with Parkinson's disease from progressive supranuclear palsy and corticobasal syndrome.

.

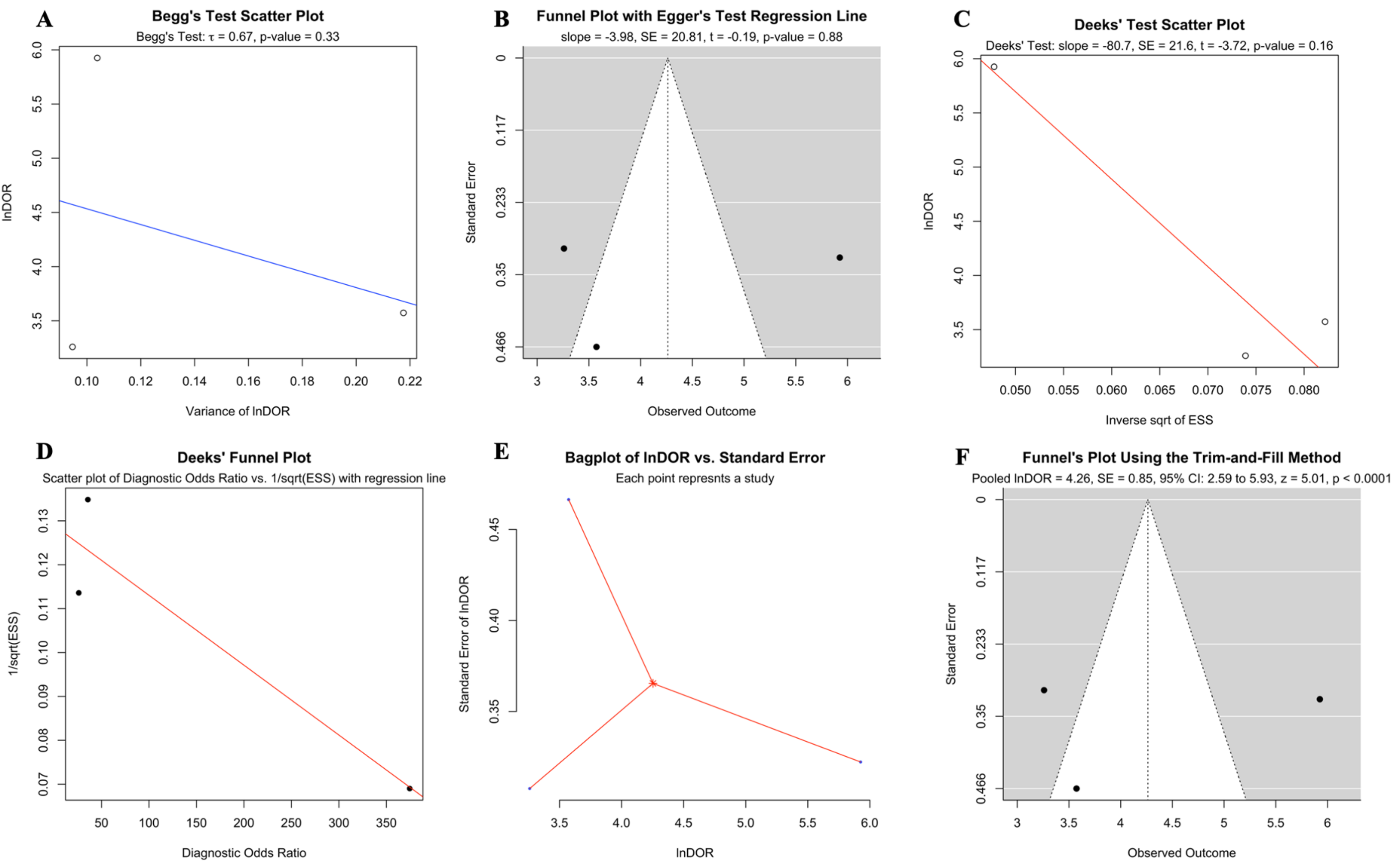

FIGURE S5. Publication bias assessed using **(A)** Begg's correlation, **(B)** Egger's regression, **(C)** Deek's regression, **(D)** Deek's funnel plot, **(E)** A bagplot and **(F)** Funnel plot after application of the trim-and-fill method for biomarkers in putative CNS-originating EVs for the differential diagnosis of Parkinson's disease from progressive supranuclear palsy and corticobasal syndrome. Collectively, they suggested no presence of publication bias.

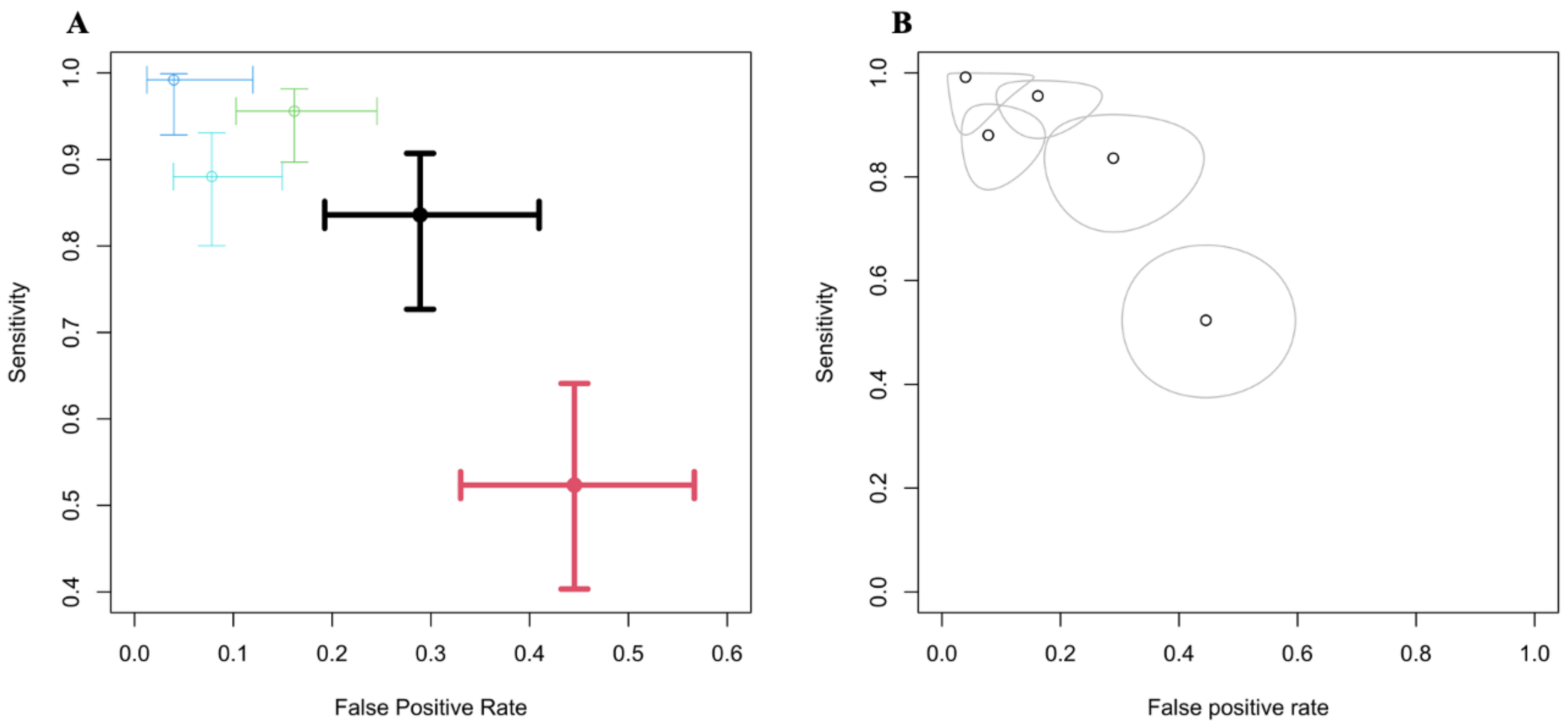

FIGURE S6. (A) Crosshair and **(B)** Receiver operating characteristics ellipse plots for studies using biomarkers in putative CNS-originating EVs for the differential diagnosis of patients with multiple system atrophy from healthy controls.

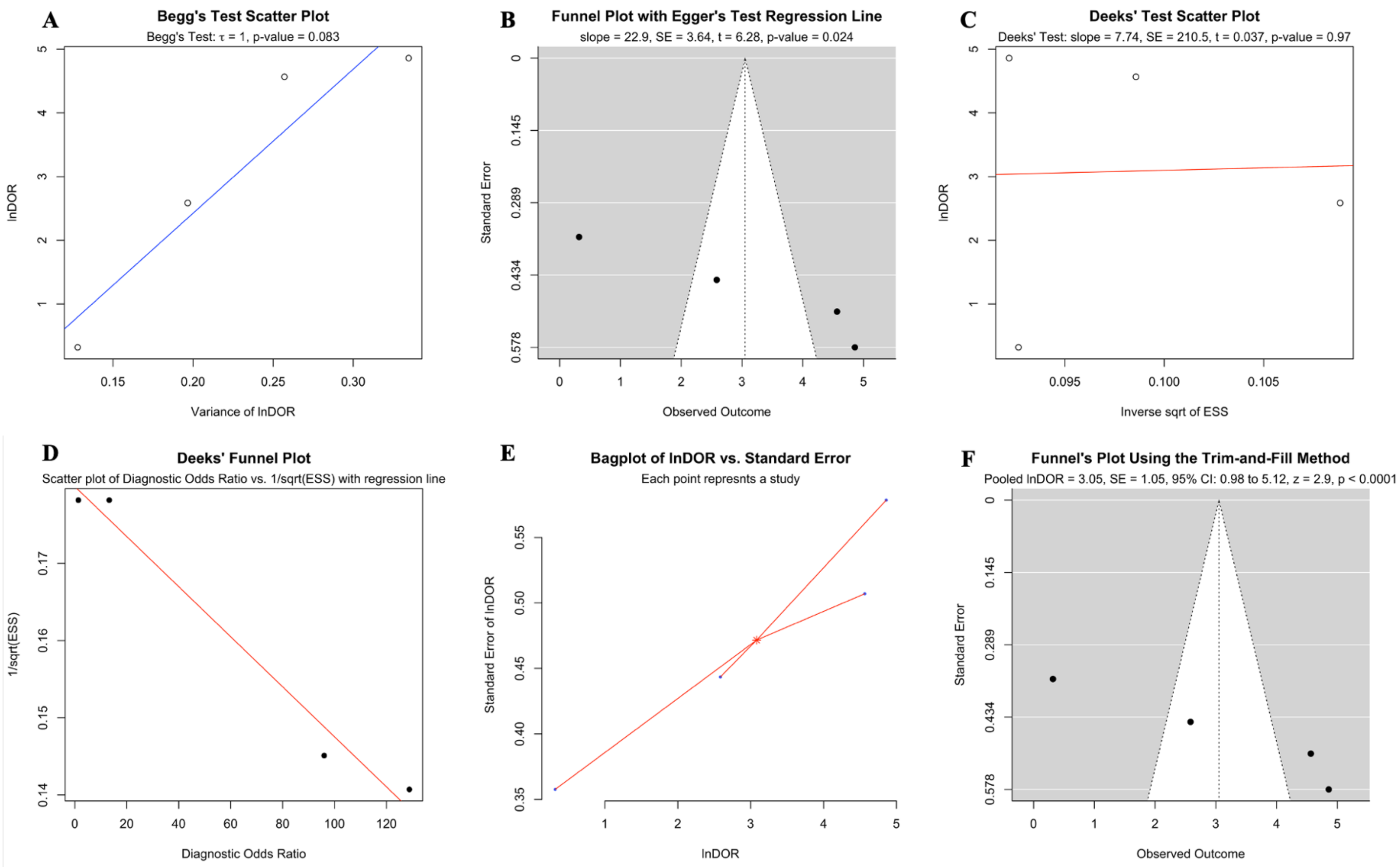

FIGURE S7. Publication bias assessed using **(A)** Begg's correlation, **(B)** Egger's regression, **(C)** Deek's regression, **(D)** Deek's funnel plot, **(E)** A bagplot and **(F)** Funnel plot after application of the trim-and-fill method for biomarkers in putative CNS-originating EVs for the differential diagnosis of multiple system atrophy from healthy controls. Collectively, they suggested no presence of publication bias.

A

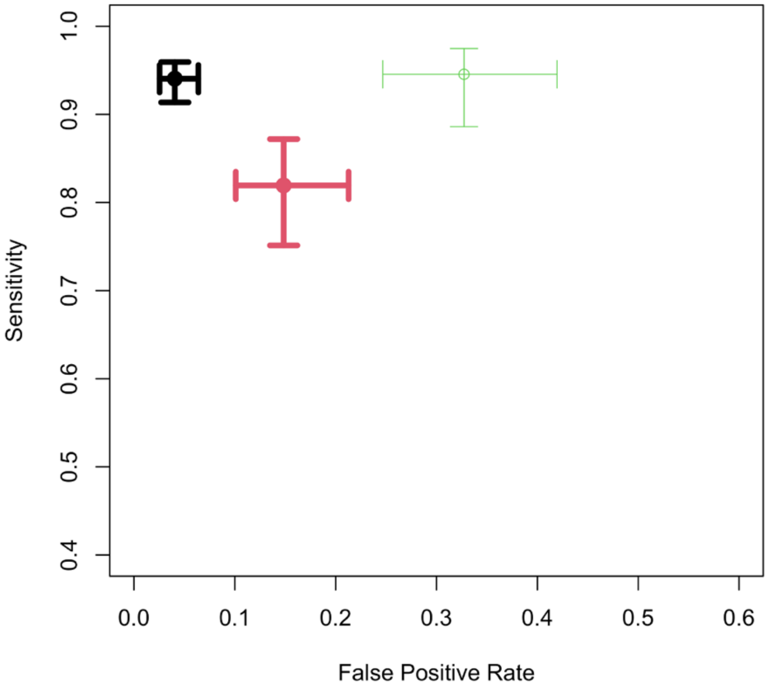

B

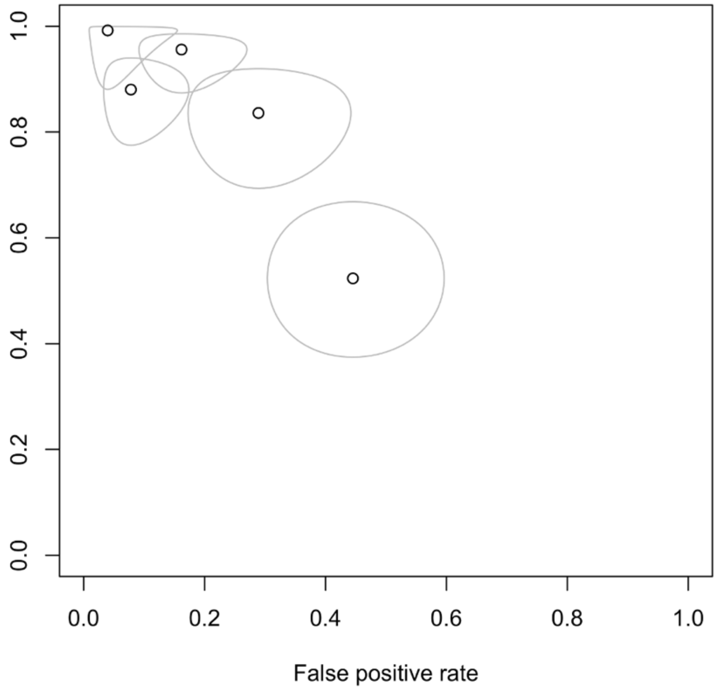

FIGURE S8. (A) Crosshair and **(B)** Receiver operating characteristics ellipse plots for studies using biomarkers in putative CNS-originating EVs for the differential diagnosis of patients with REM behavior disorder (RBD) from healthy controls.
